# Aging impairs the osteocytic regulation of collagen integrity and bone quality

**DOI:** 10.1038/s41413-023-00303-7

**Published:** 2024-02-26

**Authors:** Charles A. Schurman, Serra Kaya, Neha Dole, Nadja M. Maldonado Luna, Natalia Castillo, Ryan Potter, Jacob P. Rose, Joanna Bons, Christina D. King, Jordan B. Burton, Birgit Schilling, Simon Melov, Simon Tang, Eric Schaible, Tamara Alliston

**Affiliations:** 1 Department of Orthopaedic Surgery, University of California, San Francisco, CA 94143 USA; 2 UC Berkeley/UCSF Graduate Program in Bioengineering, San Francisco, CA 94143 USA; 3 Buck Institute for Research on Aging, Novato, CA 94945 USA; 4 Washington University in St Louis, Department of Orthopedics, St. Louis, MO 63130 USA; 5 Advanced Light Source, Lawrence Berkeley National Laboratory, Berkeley, CA 94720 USA

**Keywords:** Bone quality and biomechanics, Bone

## Abstract

Poor bone quality is a major factor in skeletal fragility in elderly individuals. The molecular mechanisms that establish and maintain bone quality, independent of bone mass, are unknown but are thought to be primarily determined by osteocytes. We hypothesize that the age-related decline in bone quality results from the suppression of osteocyte perilacunar/canalicular remodeling (PLR), which maintains bone material properties. We examined bones from young and aged mice with osteocyte-intrinsic repression of TGFβ signaling (*TβRII*^*ocy−/−*^) that suppresses PLR. The control aged bone displayed decreased TGFβ signaling and PLR, but aging did not worsen the existing PLR suppression in male *TβRII*^*ocy−/−*^ bone. This relationship impacted the behavior of collagen material at the nanoscale and tissue scale in macromechanical tests. The effects of age on bone mass, density, and mineral material behavior were independent of osteocytic TGFβ. We determined that the decline in bone quality with age arises from the loss of osteocyte function and the loss of TGFβ-dependent maintenance of collagen integrity.

## Main

Age-related bone fragility is the consequence of changes in material and molecular mechanisms that control both bone mass and bone quality over a lifetime. The loss of bone mass alone is not entirely predictive of fragility fracture in aged populations.^1,2^ For example, approximately half of age-related hip fractures in women occur in individuals with clinically normal bone mass,^2^ implicating a bone quality deficit. Nevertheless, diagnostics and treatments for poor bone quality are almost entirely unavailable, mainly due to a lack of mechanistic understanding of how bone quality is regulated with age.

Bone quality incorporates several related factors operating from the macroscale, such as bone geometry, to the micro- and nanoscales, such as trabecular microarchitecture, microdamage, and bone matrix material properties, composition, and organization.^3,4^ These factors combine across multiple length scales to determine bone toughness, or fracture resistance, which is independent of bone mass and which deteriorates with age, resulting in bone fragility. Bone quality should be examined at the micron to nanometer scales, that is, at cellular length scales, where the material consequences of biological stimuli, such as aging, sex hormones, disease or therapeutic interventions, are most direct and sensitive.^5–7^ Investigations at these length scales have already identified age-dependent changes in collagen crosslinking and other factors that compromise bone material properties.^8,9^ Although the effect of age on bone cells and the bone matrix is becoming clearer,^10–13^ the lack of understanding of the mechanisms by which these cellular changes cause material changes that toughen or embrittle bone remains a fundamental gap.

Important insight into the biological mechanisms controlling bone mass and bone quality has come from the analysis of transforming growth factor beta (TGFβ), which regulates the function of each cell type in the bone remodeling unit.^3,14–17^ Genetic alterations that dysregulate TGFβ signaling within the skeleton contribute to the increased bone fragility in osteogenesis imperfecta and Camurati-Englemann disease.^18,19^ TGFβ ligand is deposited within the bone matrix in a latent form and is activated in the acidic osteoclast microenvironment, as well as in response to other stimuli.^20^ Once activated, TGFβ binds receptors on multiple cellular targets, including osteocytes, osteoclasts, and osteoblasts, to balance bone resorption and formation.^21–25^ Age-related changes in TGFβ signaling in the skeleton are thought to uncouple osteoclast and osteoblast function, influencing bone loss.^23^ Previously, we found that osteocytic TGFβ was implicated in the control of bone quality, independent of bone mass. TGFβ regulates perilacunar/canalicular remodeling (PLR),^26,27^ a homeostatic process in which osteocytes dynamically resorb and then replace the bone matrix surrounding the osteocyte lacunar/canalicular network (LCN). The activation of PLR, for example, in bone from lactating mice, stimulates the PLR expression of proteases such as *Mmp13*, *Mmp14*, and *Ctsk* and acidifying enzymes such as ATP6v0d2 and CA2,^26–29^ as well as the expansion of perilacunar and pericanalicular spaces. Conversely, suppression of PLR, as in bone deficient in MMP13 or YAP/TAZ^30,31^ or bone from glucocorticoid-treated mice,^32,33^ causes coordinated repression of PLR proteases, degeneration of the LCN, and decreased bone quality. The disruption of osteocyte-intrinsic TGFβ signaling utilizing the *DMP1-Cre*^*+/-*^;*TβRII*^*fl/fl*^ (*TβRII*^*ocy−/−*^) mouse model led to PLR suppression, which resulted in reduced protease expression, LCN degeneration, and severe bone quality defects without significant changes in cortical bone mass.^26^ Several aspects of the male *TβRII*^*ocy−/−*^ bone phenotype recapitulate hallmarks of aging bone, including poor bone quality and a disorganized LCN.^34,35^ However, the biological mechanism responsible for the age-related loss of bone quality and the role of TGFβ or PLR in this process remain unknown.

The unique hardness and toughness of bone are derived from its composite mineral and organic constituents, especially hydroxyapatite and collagen. Both are susceptible to changes with age that contribute to the age-related decline in bone quality. Aged bone has increased mineral density and alterations in the tightly controlled size, shape, heterogeneity, and organization of hydroxyapatite crystals, all of which impact bone material quality.^36,37^ Collagen is an important determinant of bone toughness, its ability to absorb energy prior to fracture. Collagen undergoes posttranslational changes, including nonenzymatic and enzymatically catalyzed modifications and crosslinks that influence its self-assembly, structural stability, and function. In adolescent and young adult bone, the major crosslinked species are enzymatically controlled, but with age, nonenzymatic crosslinks dominate and cause stiffness in aged bone.^11,38–43^ Although these age-dependent changes in bone mineral and collagen clearly contribute to bone fragility, the cellular and molecular mechanisms by which homeostatic mineralization and collagen crosslinking are maintained or disrupted with age remain unclear.

Therefore, to identify the biological mechanisms responsible for the decline in bone quality in aged bone, we used the *TβRII*^*ocy−/−*^ mouse model with an osteocyte-intrinsic defect that suppresses PLR. Since bones from young female *TβRII*^*ocy−/−*^ mice do not exhibit the LCN degeneration or bone quality defects observed in young male *TβRII*^*ocy−/−*^ mice, we further sought to determine the role of osteocytic TGFβ signaling in the sexually dimorphic susceptibility to bone quality loss with age. Thus, by aging both male and female *TβRII*^*ocy−/−*^ bone alongside Cre-negative controls, we were able to examine the roles of age, osteocytic TGFβ signaling, and PLR in the control of bone quality. By combining this investigative scheme with multiscale materials science approaches with cellular and mechanical techniques, the extent to which these effects result from changes in the material performance of collagen or mineralization and the extent to which they rely on osteocytes can be ascertained.

## Results

### Depletion of skeletal TGFβ signaling with age

**Fig. 1:**
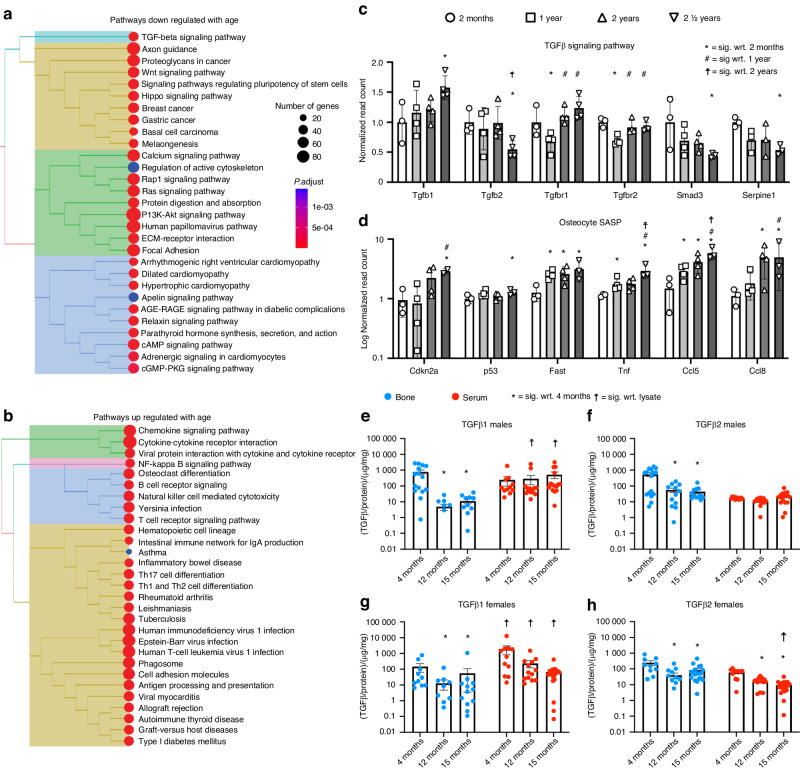
Bone aging is associated with reduced TGFβ signaling and increased cellular senescence. Unbiased hierarchical pathway clustering of KEGG pathways for the whole transcriptome of male C57BL/6 (WT) mouse bone showed several pathways that were downregulated (**a**) or upregulated (**b**) with age, including the downregulated TGFβ signaling pathway. Analysis of individual genes at the transcriptional level showed reduced levels of several genes in the TGFβ signaling pathway (**c**) and elevated levels of markers of osteocyte senescence and the senescence-associated secretory phenotype (SASP) (**d**) in bone with age. Multiplexed ELISA of homogenized bone lysate (blue) showed age-related reductions in TGFβ ligand levels in the bone of both males and females (**e**–**h**). The circulating TGFβ ligand levels in blood serum (red) remained stable over time in males but showed decreases in females. *P* < 0.008 using Student’s t test with the Bonferroni correction with respect to (wrt.) 2 months (*), 12 months (^#^), and 24 months (☨), *n* = 3-4 per group. **e**–**h** *P < 0.008 wrt. 4 months from the same source (bone lysate or serum), ^☨^*P* < 0.008 wrt. The age^-^matched opposite tissue source (lysate vs. serum) in post hoc pairwise Fischer’s LSD test after two-way ANOVA, *n* = 12–15 per group

To elucidate the molecular mechanisms responsible for the age-related decline in bone quality, we first performed RNA sequencing on cortical bone from aged male C57/Bl6 (WT) mice. Unbiased hierarchical clustering of differential gene expression with age in bone (Fig. 1a, b) revealed several important profiles. Pathways that are downregulated with age include the TGFβ, Wnt, parathyroid hormone, PI3K-Akt, and calcium signaling pathways, as well as processes associated with ECM remodeling and adhesion (Fig. 1a). Because of the known role of TGFβ signaling in the control of bone quality,^14,15,17,26^ we further examined the effect of age on genes involved in the TGFβ signaling pathway. The levels of mRNA encoding TGFβ ligands, receptors, and effectors changed with age; some were induced, and others were repressed (Fig. 1c). Transcriptional readouts for the TGFβ inducible target gene *Serpine1* are significantly repressed in 2 ½-year-old WT bone. The upregulated pathways include those involved in osteoclast differentiation and NF-κΒ signaling, as well as markers of osteocyte senescence or the senescence-associated secretory phenotype (SASP), including *Cdkn2a* (p16), *Fasl*, and *Tnf*, which were dramatically upregulated with age, as previously reported (Fig. 1d).^12,44^ Together, these results suggest a repression of TGFβ signaling at the transcriptional level with age in normally aging bone.

To determine whether posttranscriptional mechanisms contribute to an age-dependent loss in TGFβ signaling, we evaluated the levels of TGFβ1, 2, and 3 in cortical bone lysate and serum at 4, 12, and 15 months of age. The concentrations of both TGFβ1 and TGFβ2 decreased with age in bone in both males and females (Fig. 1e–h). The only significant change in serum with age was a decrease in TGFβ2 levels in female mice. Overall, the most significant predictor of ligand abundance in serum and bone over time was age. TGFβ1 levels were higher in serum than in bone, but the TGFβ2 levels were equivalent in both compartments. Although TGFβ levels in bone did not differ between the sexes, females had 25% more circulating ligand levels than males and showed a greater percentage loss of circulating TGFβ with age than males. Thus, evidence of TGFβ signaling in bone declines with age, with transcriptional and posttranscriptional changes at multiple levels of the signaling pathway.

### Osteocyte-intrinsic TGFβ signaling impacts tissue-scale bone aging

In addition to the decline in TGFβ signaling in bone with age, bones from young mice with impaired osteocytic TGFβ signaling show signs of altered aging. Specifically, both aged WT mice and *TβRII*^*ocy−/−*^ mice, in which TGFβ receptor type II is deleted in osteocytes, exhibit poor bone quality and dysregulated osteocyte lacunocanalicular networks relative to those of young or Cre-negative controls.^26,34^ However, the role of TGFβ signaling in osteocytes and their precursors in skeletal aging is unknown*.* Also unknown are the molecular and material mechanisms by which changes to cellular function, with age or impaired TGFβ signaling, compromise bone quality. While 10 kb DMP1-Cre may cause excision in osteoblasts, previous works with *TβRII*^*ocy−/−*^ mice have not demonstrated alterations in bone mass or shape, osteoblast numbers, or osteocyte numbers, which might be anticipated if its effects were due primarily to the excision of TβRII from osteoblasts.^26,27,34^ The predominance of effects attributed to osteocyte-intrinsic interruptions to TGFβ signaling in these mice led to their use as a model to dissect the osteocyte-intrinsic role of TGFβ signaling in the mechanical and biological manifestations of bone aging.

**Fig. 2:**
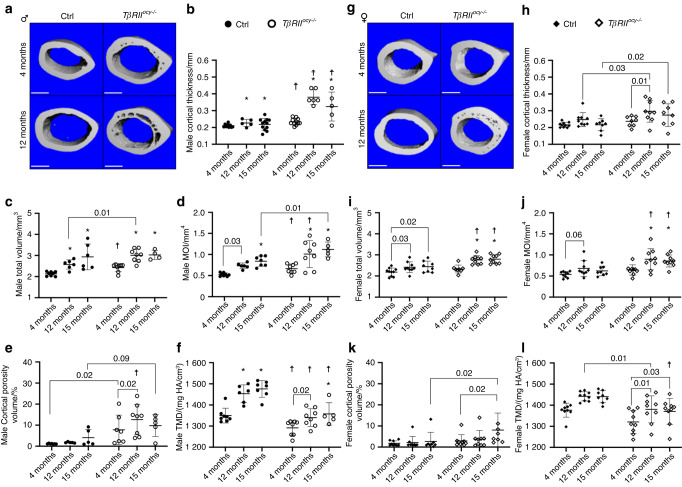
Accelerated bone structure changes in *TβRII*^*ocy−/−*^ cortical bone. μCT of male *TβRII*^*ocy−/−*^ bone (**a**) revealed several signs of an accelerated age-like phenotype, including increased cortical thickness **(b**), overall bone volume (**c**) and MOI (**d**). Male *TβRII*^*ocy−/−*^ bone also displayed the development of intracortical porosities (**a**, **e**), resulting in trabecularization of the cortex. *TβRII*^*ocy−/−*^ males also exhibited lower bone mineral density (TMD) (**f**) than Cre-negative controls at all ages but did show expected age-related increases in bone density. Females (**g**) did not show any genotype-specific differences in pairwise comparisons to Cre-negative controls until 1 year, when they begin to display small, nonsignificant increases in thickness (significant main effect of genotype and age) (**h**) and significant increases in bone volume and MOI (**i**, **j**). Similar to males, female *TβRII*^*ocy−/−*^ bone developed cortical porosity (significant main effect of genotype); however, these differences were not significant in pairwise comparisons at the corrected alpha levels (**k**). Similar to that in males, aged female *TβRII*^*ocy−/−*^ bone displayed lower tissue mineral density than the Cre-negative controls (**l**). *P < 0.007 wrt. 4 months Cre-negative control, ^☨^*P* < 0.007 wrt^.^ Age-matched within-genotype group in the 7-way Bonferroni corrected post hoc pairwise Fischer’s LSD test after two-way ANOVA. *n* = 8–12 per group

Microcomputed tomography revealed genotype-dependent differences in the effect of age on cortical and trabecular bone. In males (Fig. 2a) at 4 months, *TβRII*^*ocy−/−*^ cortical bone was thicker, larger, and more irregularly shaped than that of age-matched controls, with increased cortical thickness, total volume, and moment of inertia (MOI) (Fig. 2b–d). Both the control and *TβRII*^*ocy−/−*^, mice showed the anticipated progressive expansion of cortical bone with age. Other important genotype-dependent differences in cortical bone were observed in cortical porosity and tissue mineral density (TMD) (Fig. 2e, f). Intracortical pores were observed as early as 4 months in male *TβRII*^*ocy−/−*^ bone, even though no such pores were detected until 15 months of age in Cre-negative controls (Fig. 2a, e). Finally, although both groups show the expected age-related increase in TMD,^45^ *TβRII*^*ocy−/−*^ males displayed lower TMD at all ages, which was consistent with previous findings.^26^^,^^27^ Although all μCT parameters reveal an altered aging trajectory for male *TβRII*^*ocy−/−*^ bone only the cortical thickness presented a significant interaction term between aging and genotype, implying that the change in cortical thickness with age is dependent on the genotype. All other parameters, except cortical porosity, presented significant main effects for both age and genotype by two-way ANOVA with no interactions. Cortical porosity was the only μCT parameter to display only genotype main effects. Interestingly, direct comparison of Cre-negative control males at 15 months of age and *TβRII*^*ocy−/−*^ males at 4 months showed no significant differences in post hoc pairwise comparisons for cortical thickness, MOI, total volume, or cortical porosity, while direct tests for statistical similarity (overlapping 95% confidence intervals) also showed these two groups to be statistically indistinguishable from one another (Fig. S1).

As previously reported, young *TβRII*^*ocy−/−*^ female bone was indistinguishable from controls by μCT (Fig. 2g).^27^ However, at 12 and 15 months, *TβRII*^*ocy−/−*^ female bones exhibited slight increases in cortical thickness and significantly elevated MOI and total volume compared to their age-matched controls (Fig. 2h–j), similar to the enlargement observed in males. In fact, although post hoc analysis falls short in showing certain pairwise significant changes, for instance, in cortical thickness, the significant main effects of genotype, along with the significant main effect of age, imply that with age, even female *TβRII*^*ocy−/−*^ bones show meaningful changes in bone morphology that are dependent on TGFβ signaling.

With age, *TβRII*^*ocy−/−*^ female bone shows a trend toward the accumulation of intracortical pores (Fig. 2k), which was not detected in female control bone at these times. *TβRII*^*ocy−/−*^ female bone showed similar changes to aging *TβRII*^*ocy−/−*^ males but to a lesser degree. Similar to males, this is the only parameter observed by μCT to exhibit a significant main effect of genotype alone without an accompanying significant effect of age. *TβRII*^*ocy−/−*^ females resembled *TβRII*^*ocy−/−*^ males in TMD (Fig. 2l) to a lesser degree and were not significantly different from Cre-negative controls until 15 months of age in post hoc pairwise comparisons at each age.

Within the trabecular compartment (Table 1), the Cre-negative controls showed anticipated losses of trabecular bone with age.^46^ As previously reported, young male *TβRII*^*ocy−/−*^ bone had increased BV/TV and mineral density (TB. TMD) compared to the Cre-negative controls,^16,26,27^ but no differences were observed in trabecular number, thickness, or spacing. The elevated BV/TV in young *TβRII*^*ocy−/−*^ males tempered the age-related trabecular bone losses seen in the controls. The effect of age on female trabecular bone was more pronounced than that on male trabecular bone. In contrast to the persistence of male *TβRII*^*ocy−/−*^ trabecular bone with age, female trabecular bone for both genotypes exhibited similar age-related losses. In summary, the effect of osteocytic TGFβ deficiency on bone structure is more severe in males than in females and is consistent with an altered aging phenotype. While some bone structure parameters are unaffected by age in *TβRII*^*ocy−/−*^ mice (e.g., male trabecular BV/TV), others are more sensitive to aging than controls (e.g., male cortical thickness).

**Table 1:** Male *TβRII*^*ocy−/−*^ trabecular bone displays resistance to aging in μCT parameters

Sex	Genotype	Age/month	BV/TV	TB.TMD/(mg HA/cm^3^)	CD per mm^3^	TB.N per mm	TB.Sp/mm	Tb.Th/mm
M	TβRII Ctrl	4	0.16 ± 0.04	1 044.81 ± 46.54	235.35 ± 48.24	5.23 ± 0.37	0.19 ± 0.02	0.045 ± 0.005
		12	0.07 ± 0.07^*^	1 112.88 ± 31.58^*^	71.38 ± 118.28^*^	3.11 ± 1.12^*^	0.35 ± 0.10^*^	0.049 ± 0.006
		15	0.05 ± 0.05^*^	1 143.29 ± 41.13^*^	40.78 ± 44.10^*^	2.65 ± 0.71^*^	0.40 ± 0.10^*^	0.053 ± 0.008
	*TβRII*^*ocy−/−*^	4	0.22 ± 0.09	1 110.76 ± 8.43^☨^	204.19 ± 61.45	5.29 ± 0.86	0.18 ± 0.04	0.050 ± 0.008
		12	0.18 ± 0.05^☨^	1 160.49 ± 30.16	146.04 ± 89.69^*,☨^	4.24 ± 0.60^☨^	0.23 ± 0.03^☨^	0.052 ± 0.009
		15	0.17 ± 0.06	1 125.68 ± 64.34	100.11 ± 67.18^*^	3.33 ± 0.52^*^	0.31 ± 0.05^*^	0.063 ± 0.020
F	TβRII Ctrl	4	0.11 ± 0.03	1 125.09 ± 16.84	115.69 ± 38.85	3.76 ± 0.32	0.27 ± 0.03	0.050 ± 0.002
		12	0.08 ± 0.09	1 205.10 ± 57.69^*^	12.83 ± 11.95^*^	2.07 ± 0.45^*^	0.51 ± 0.10^*^	0.069 ± 0.033
		15	0.06 ± 0.05	1 195.93 ± 53.49^*^	16.73 ± 21.98^*^	1.77 ± 0.27^*^	0.59 ± 0.09^*^	0.063 ± 0.023
	*TβRII*^*ocy−/−*^	4	0.18 ± 0.10	1 161.28 ± 28.78	97.51 ± 55.42	3.65 ± 1.03	0.30 ± 0.13	0.072 ± 0.023
		12	0.09 ± 0.09	1 192.31 ± 35.88	25.91 ± 19.64^*^	2.20 ± 0.51^*^	0.49 ± 0.10^*^	0.064 ± 0.022
		15	0.09 ± 0.09	1 190.79 ± 46.77	22.01 ± 30.24^*^	1.86 ± 0.37^*^	0.60 ± 0.11^*^	0.071 ± 0.025

The trabecular bone parameters measured by μCT primarily showed genotype-dependent differences in males, where male *TβRII*^*ocy−/−*^ mice resisted the anticipated age-related decline in BV/TV and TB. BMD, with a tempered loss in connective density (CD). Control males and females of both genotypes experienced age-related bone loss. *P < 0.007 wrt. 4 months within genotype control, ^☨^*P* < 0.007 wrt^.^ age-matched genotype control in 7-way Bonferroni corrected post-hoc pairwise Fischer’s LSD test after two-way ANOVA. *n* = 8–12 per group

### Whole bone stiffness and strength are sensitive to age and limited TGFβ signaling

**Fig. 3:**
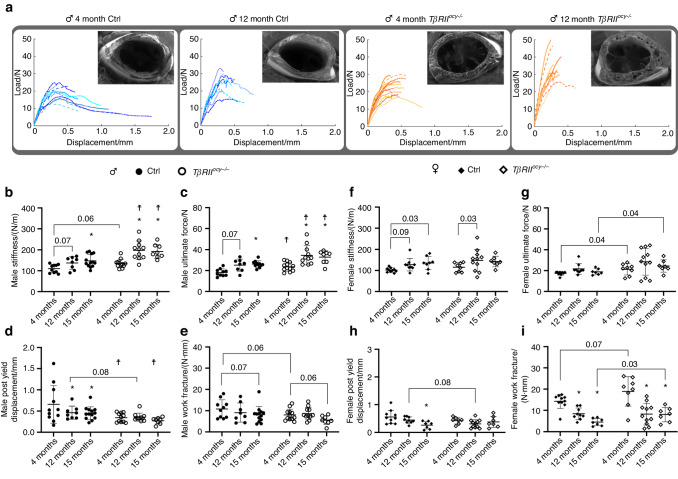
Aging and *TβRII*^*ocy−/−*^ bone demonstrates reduced bone toughness despite increases in tissue stiffness and strength. **a** Observation of load displacement curves and fracture surfaces via SEM from three-point bend mechanical testing of femurs from male *TβRII*^*ocy−/−*^ bone and age-matched controls showed similar fracture and failure mechanisms in *TβRII*^*ocy−/−*^ bone and aged controls compared to young control bone. The quantification of mechanical properties showed increases in tissue stiffness (**b**) and fracture force (**c**) with both age and the loss of TGFβ signaling in males. Despite increases in tissue strength, the aged controls and *TβRII*^*ocy−/−*^ bone displayed a decrease in postyield displacement (**d**), resulting in no improvement in tissue-level toughness in the work of fracture (**e**). Female *TβRII*^*ocy−/−*^ (**f**–**i**) bones did not show significant changes in three-point bend parameters in age-matched pairwise comparisons to Cre-negative controls but did show age-related changes. The ultimate force and fracture work did display an important main effect of genotype. Both Cre-negative controls and *TβRII*^*ocy−/−*^ females showed age-related decreases in the work of fracture (**i**). *P < 0.007 wrt. 4 months Cre-negative control, ^☨^*P* < 0.007 wrt^.^ age-matched-within-genotype group in a 7-way Bonferroni-corrected post hoc pairwise Fischer’s LSD test after two-way ANOVA. *n* = 8–12 per group

Three-point bending mechanical tests were performed to determine alterations in bone strength and quality in *TβRII*^*ocy−/−*^ mice with age. Load‒displacement curves and bone fracture surfaces (insets) (Fig. 3a) revealed striking differences in failure behavior between male Cre-negative controls and *TβRII*^*ocy−/−*^ bones. Observation of the load‒displacement curves shows that young male Cre-negative controls display, on the whole bone scale, extended postyield deflection and jagged or oblique fracture surfaces, evidence that crack deflection mechanisms were utilized to dissipate stress and increase toughness.^47^ These behaviors are less prominent in the 12-month-old Cre-negative controls and in the young *TβRII*^*ocy−/−*^ males, as indicated by the truncation of the load‒displacement curves and the occurrence of more perpendicular fracture surfaces. These altered failure behaviors are further exaggerated in 12-month-old *TβRII*^*ocy−/−*^ male bones.

Quantitative analysis confirmed the alterations in whole bone strength with age and diminished osteocytic TGFβ signaling. Consistent with prior reports,^46,48,49^ we qualitatively observed anticipated age-dependent changes, including increased stiffness and ultimate force with age (Fig. 3b, c) and decreased postyield displacement (Fig. 3d). The same trends were apparent in aging *TβRII*^*ocy−/−*^ males, but they were accelerated so that stiffness and strength, measured by ultimate force, were increased, while 4-month-old male *TβRII*^*ocy−/−*^ bones exhibited reduced postyield displacement relative to Cre-negative controls of the same age. However, postyield displacement in male *TβRII*^*ocy−/−*^ mice did not decline further with age, showing no significant differences in this parameter across 4, 12, or 15 months. The increases in stiffness and strength in male *TβRII*^*ocy−/*−^ bones offered no advantage to their material quality, as assessed by my fracture work (Fig. 3e), which did not differ significantly between genotypes at any age. Other measures of material quality from three-point bend analysis, calculated from the structural parameters normalized by the bone cross-section, including the bending modulus, yield, and ultimate stress, also showed no differences between Cre-negative controls and *TβRII*^*ocy−/−*^ mice (Fig. S2).

The mechanical testing of young female bones produced results that matched those in previous reports,^27^ with no side effect of impaired osteocytic TGFβ signaling. Female Cre-negative controls showed the anticipated decreases in postyield displacement and work-to-fracture with age.^11,50,51^ Cre-negative control and *TβRII*^*ocy−/−*^ bones did not significantly differ in whole bone stiffness, ultimate force, postyield displacement, or work of fracture at any age (Fig. 3f–i). For ultimate force and work of fracture, two-way ANOVA showed a significant main effect of genotype between *TβRII*^*ocy−/−*^ females and Cre-negative controls regardless of age, but subsequent pairwise post hoc comparisons for each independent age group failed to reach the corrected significance level. The results of these complex macromechanical and μCT analyses reflect longitudinal responses to altered osteocytic TGFβ signaling with age.

### Relationship of age and osteocytic TGFβ signaling in nanoscale material mechanisms

**Fig. 4:**
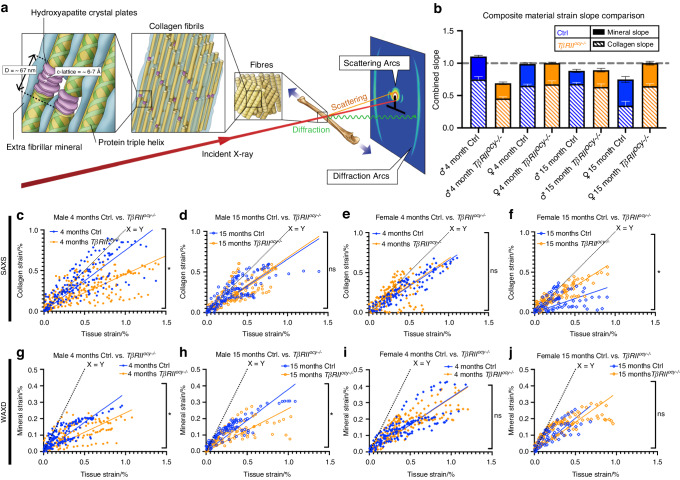
Synchrotron SAXS/WAXD shows altered collagen and mineral behavior in *TβRII*^*ocy−/−*^ bone during in situ tensile testing. **a** Synchrotron-generated X-ray exposure during tensile testing of the forearm bones of *TβRII*^*ocy−/−*^ and Cre-negative controls at multiple ages generates signals unique to organic collagen (SAXS) and the inorganic mineral components (WAXD) of bone based on their hierarchical size, order, and structure. The summation of composite strains (**b**) reveals deficiencies in the ability of collagen and minerals to carry tissue-level strains in young, male *TβRII*^*ocy−/−*^ and aged, female Cre-negative controls. Compared to Cre-negative controls, young *TβRII*^*ocy−/−*^ males showed a deficiency in collagen strain capacity (**c**) that recovered with age (**d**). Female *TβRII*^*ocy−/−*^ mice did not show this weakness at young ages (**e**). With age, female Cre-negative controls showed a decrease in collagen strain, while *TβRII*^*ocy−/−*^ did not, resulting in significant differences in collagen strain between these two groups (**f**). The mineral strains in young male bones showed genotype-dependent differences (**g**) that did not change with age (**h**), while females did not show genotype differences at either age studied (**i, j**). *P < 0.012 5 for 4-way Bonferroni correction in comparisons of regression slopes in an extra sum-of-squares F test. *n* = 5-8 per group

Since the complexity of tissue-scale analyses and the gross alterations to bone morphology obscure the role of osteocytic TGFβ signaling in regulating bone material properties with age, we pursued analyses at smaller scales to interrogate changes occurring in the principal components of bone: collagen and mineral. Leveraging differences in the nanoscale periodic structure of collagen and minerals, high-intensity monochromatic synchrotron-generated X-rays can discriminate their material behavior in in situ tensile mechanical tests (Fig. 4a).^11,52,53^ When incident X-rays pass through bone, the nanoscale molecular repeats of collagen fibrils scatter X-rays at a shallow angle, while the Angstrom scale spacings of the mineral lattice diffract X-rays at a wider angle. The resulting separation between small-angle and wide-angle X-ray (SAXS and WAXD) signals was captured, and changes in the signals during tensile testing allowed the strain to be calculated independently for the collagen and mineral phases. Whole tissue strain was calculated using visible light CCD images of the bone. Ideally, the sums of the strain slope fits (Table 2) from bone constituent materials, mainly collagen and mineral, would be approximately 1, indicating that 100% of the tissue strain is captured by the nanomaterials observed. This relationship is apparent in young control male and both control and *TβRII*^*ocy−/−*^ female bone (Fig. 4b), where collagen and mineral slopes sum to approximately 1, indicating a stable mechanical composite in these cases. The appearance of young male Cre-negative bone to have a summed slope >1 likely results from the collection of data on separate contralateral bones instead of simultaneous collection on a single sample. However, a slope <1, as in young, male *TβRII*^*ocy−/−*^ bone, can indicate losses to collagen, mineral, or other unobserved material-associated strains and a disconnect between material-scale and tissue-scale strain. The relative distribution of strain between the organic and mineralized components shows sex and genotype-dependent differences with age (Fig. S3).

**Table 2:** Slopes of composite material strain vs. tissue strain

	Collagen Slope		Mineral Slope	
	4 month	15 month	4 month	15 month
Ctrl Male	0.748 2 ± 0.045 1	0.678 0 ± 0.022 0	0.356 1 ± 0.019 1	0.383 2 ± 0.023 2
*TβRII*^*ocy−/−*^ Male	0.453 9 ± 0.027 2^☨^	0.631 9 ± 0.047 8*	0.235 2 ± 0.020 7^☨^	0.260 5 ± 0.028 6^☨^
Ctrl Female	0.650 0 ± 0.053 0	0.342 7 ± 0.067 6*	0.337 3 ± 0.019 0	0.405 3 ± 0.045 9
*TβRII*^*ocy−/−*^ Female	0.673 5 ± 0.045 1	0.644 8 ± 0.037 5^☨^	0.327 9 ± 0.015 4	0.356 7 ± 0.029 3

Fits of scatter plots of material strains as captured by synchrotron-generated X-ray exposure versus tissue scale strains of whole bones captured by CCD camera and DIC analysis during uniaxial tensile testing show the relative proportion of tissue strain carried by each composite material. * = *P* < 0.012 5 wrt. age within genotype ↔, ☨ = *P* < 0.012 5 wrt. genotype at the same age ↕

Analysis of the collagen and mineral material behavior yielded insight into the complex role of osteocytic TGFβ signaling in bone aging. SAXS revealed that collagen in young male *TβRII*^*ocy−/−*^ bone is less able to carry strain than that in age-matched control bone (Fig. 4c). By 15 months, the collagen strain in male *TβRII*^*ocy−/−*^ bone was statistically indistinguishable from that in the control bone (Fig. 4d), indicating a role for osteocyte-intrinsic TGFβ signaling in the age-related decline in the material quality of collagen.

The sexually dimorphic control of bone quality was also apparent in the SAXS analyses of both Cre-negative control and *TβRII*^*ocy−/−*^ bone. Collagen in young female *TβRII*^*ocy−/−*^ bone did not differ from that in the controls, which was consistent with the lack of other genotype-dependent differences in young females (Fig. 4e). Collagen strains in control aged female bone were almost half that of their younger counterparts, and therefore, the age-related loss of collagen strain is more severe in female than in male bone (Fig. S3a, c). In contrast, the collagen strain remained constant in *TβRII*^*ocy−/−*^ female bone with age, resulting in a significant difference in collagen strain between control and *TβRII*^*ocy−/−*^ female bone with age (Fig. 4f).

An evaluation of the spread of the scattering and diffraction peaks from X-ray scans by the second Legendre coefficient (P2) showed that prior to testing, collagen organization was lower by both age and TGFβ deficiency (Fig. S4A, B). Additionally, the change in P2 throughout mechanical testing revealed a small but significant inability for collagen in young *TβRII*^*ocy−/−*^ males to realign (increase in P2) with applied strain, suggesting that collagen fibrils may slide against each other instead of engaging in straining mechanisms (Fig. S4C–F), a behavior not observed in other comparisons.

The WAXD results for contralateral bones showed genotype-dependent but not time-dependent changes in mineral strains in males, while the mineral material in female bone was unaffected by either time or osteocytic deficiency in TGFβ signaling. Young male *TβRII*^*ocy−/−*^ bones once again showed significant losses to material strain capacity compared to that of Cre-negative controls (Fig. 4g), and these differences persisted with age (Fig. 4h). Female bone did not show significant genotype-dependent differences in mineral strains at either age (Fig. 4i, j). Together, the SAXS and WAXD results highlight distinct roles in male and female bone for osteocytic TGFβ signaling in the regulation of bone’s constituent materials and their behavior.

### Loss of TGFβ signaling in young male osteocytes regulates cellular aging

**Fig. 5:**
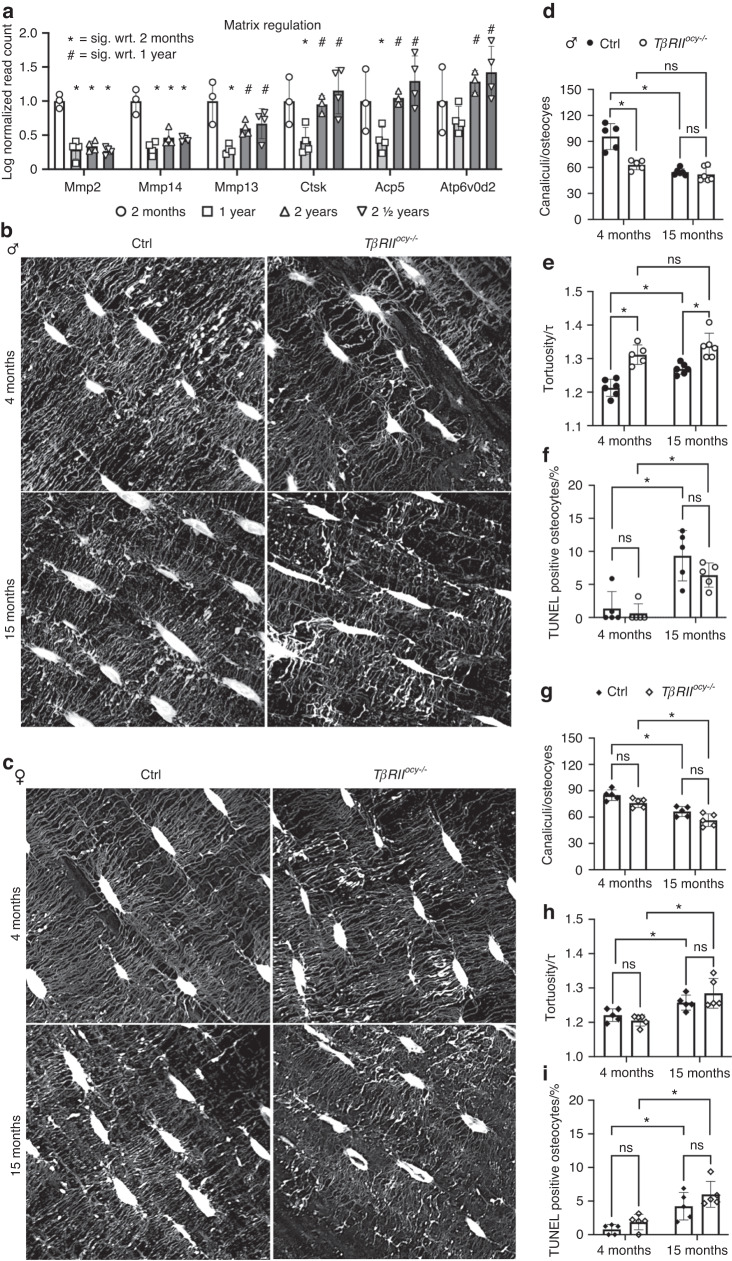
Osteocyte LCN integrity shows coordinated control by age and osteocytic TGFβ signaling. Osteocyte aging in WT C57/Bl6 control bone shows suppressed PLR, with an early and prolonged decline in the expression of MMP2 and MMP14, and dynamic expression of other factors implicated in PLR (**a**) analyzed with Fischer’s LSD test after one-way ANOVA. Visualization of the osteocyte LCN via fluorescence confocal microscopy in *TβRII*^*ocy−/−*^ and Cre-negative controls with age (**b, c**) shows a genotype-specific degeneration of the LCN in males, with losses to canaliculi/osteocyte (**d**) and increased canalicular tortuosity (**e**) in *TβRII*^*ocy−/−*^ bone that does not worsen with age. Cell death in males increased only with age (**f**). Females showed age-related changes in these parameters (**h**–**i**) in both genotypes. *P < 0.012 5 for 4-way Bonferroni corrected post hoc pairwise Fischer’s LSD test after two-way ANOVA. *n* = 4–5 per group

Given that the losses to bone quality extend to the nanoscale, we evaluated several parameters in osteocytes, the cells responsible for the upkeep and management of bone material. We previously established that the suppression of osteocytic PLR in male *TβRII*^*ocy−/−*^ bone was responsible for the loss of bone quality.^26^ Therefore, we examined the effect of aging on the expression of genes implicated in PLR^26^ and the dependence of age-related LCN degeneration on osteocytic TGFβ signaling. The expression of several genes implicated in osteocyte PLR showed age-related changes in WT control cortical bone (Fig. 5a). The expression of the collagenases Mmp2 and Mmp14 showed significant and lasting downregulation from 2 months through 2 ½ years of age, while Mmp13 showed a repression of transcriptional activity by 1 year, with some amount of recovery by 2 and 2 ½ years. Additionally, the expression of Ctsk, Acp5, and genes implicated in acidification followed similar patterns to Mmp13 in expression, showing repression at 1 year of age but full recovery by 2 ½ years.

The age-related suppression of PLR protease expression and TGFβ signaling (Fig. 1c) corresponds to the previously reported degeneration of the LCN with age and osteocytic TGFβ deficiency.^26,34^ To determine the role of osteocyte-intrinsic TGFβ signaling, we examined the integrity of the osteocyte LCN within the cortical bone of young and aged *TβRII*^*ocy−/−*^ mice and their age-matched Cre-negative controls in 3D with fluorescence confocal microscopy (Fig. 5b, c). Similar to prior reports of younger *TβRII*^*ocy−/−*^ bone or bone from wild-type mice treated with a TβRI inhibitor,^26,34^ 4-month-old male *TβRII*^*ocy−/−*^ bone displayed significantly fewer canaliculi per osteocyte and increased canalicular tortuosity compared to age-matched Cre-negative controls (Fig. 5d, e). Male Cre-negative control bones displayed similar qualitative LCN changes over time, with a significant loss of canaliculi and an increase in canalicular tortuosity, which indicated LCN degeneration with age. The interaction term between aging and genotype was significant for the canalicular number (*P* = 0.000 9), highlighting the different effects of aging on the male genotypes and LCN integrity. Interestingly, the interaction for canalicular tortuosity was not significant (*P* = 0.221 7), but canalicular tortuosity did display important significant changes in age and genotype independently, indicating significant changes in both age and genotype on LCN structure. Importantly, however, *TβRII*^*ocy−/−*^ male bone showed no pairwise differences between 4 months and 15 months in either canalicular number or tortuosity, emphasizing the similarity between the early LCN dysregulation in *TβRII*^*ocy−/−*^ male bone and the time-dependent LCN degeneration in Cre-negative controls. In both Cre-negative controls and *TβRII*^*ocy−/−*^ males, osteocyte death assessed by TUNEL staining (Fig. 5f) only showed age-related increases and did not differ by genotype at either age. Combined, these observations implicate the loss of TGFβ in age-related LCN degeneration in Cre-negative control bone.

With age, both control and *TβRII*^*ocy−/−*^ females showed losses to canalicular number and increased canalicular tortuosity compared to those of their young Cre-negative controls, but no differences in these values was observed between the genotypes at the same ages (Fig. 5g, h). These differences were not reliant on osteocyte death, since the increase in osteocyte TUNEL staining with age was unaffected by genotype in either male or female bone (Fig. 5f, i). Therefore, the age-related decline in LCN integrity in male, but not female, bone relies on osteocytic TGFβ signaling.

### Posttranslational modifications in *TβRII*^*ocy−/−*^ bone collagen occur with altered enzymatic function

Given the role of osteocyte-intrinsic TGFβ signaling in the age-related decline in LCN and collagen integrity, we took several approaches to examine collagen posttranslational modifications (PTMs) and the osteocytic mechanisms controlling them. Collagen PTMs include those formed by lysyl oxidase (LOX) and prolyl 3 hydroxylases (P3H), help to direct collagen self-assembly, crosslinking, and fibril structure.^54–58^ Accordingly, collagen PTMs underpin bone quality and contribute to fragility in aging, diabetes, and osteogeneses imperfecta, where specific PTMs are suppressed.^55,57,59^

**Fig. 6:**
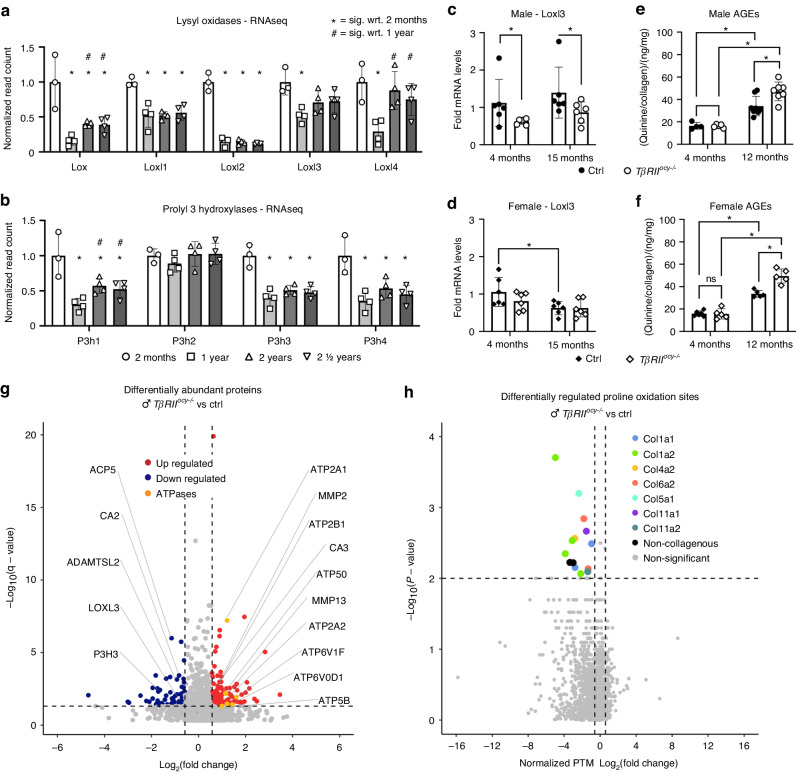
Collagen quality is impacted by both age and osteocytic TGFβ signaling. RNA sequencing of C57/Bl6 (WT) mouse cortical bone showed age-dependent transcriptional repression of several lysyl oxidase (**a**) and prolyl 3 hydroxylase (**b**) isoforms responsible for posttranslational enzymatic collagen modifications *P < 0.008 wrt. 2 months, ^#^*P* < 0.008 wrt. 1 yr. in 6-way Bonferroni corrected post hoc pairwise Fischer’s LSD test after one-way ANOVA, *n* = 3–4 per group. RT‒qPCR from *TβRII*^*ocy−/−*^ and Cre-negative control bone revealed significant repression of Loxl3 mRNA in male (**c**) *TβRII*^*ocy−/−*^ regardless of age, while females showed only an age-related decrease in Cre-negative controls (**d**). Quantification of nonenzymatic collagen crosslinking in the form of fluorescent advanced glycation end products (AGEs) shows an accelerated rate of accumulation in male and female *TβRII*^*ocy−/−*^ bone with age compared to that in the Cre-negative controls (**e**, **f**). *P < 0.012 5 in 4-way Bonferroni correction after two-way ANOVA, *n* = 5–6 per group. Proteomic analysis of male *TβRII*^*ocy−/−*^ femurs (**g**) identified 2015 quantifiable proteins, each with at least 2 unique peptides, and revealed the significant differential regulation (q-value < 0.05, and absolute Log_2_(fold-change) > 0.58), of 186 proteins. Of these, proteins involved in matrix modification and matrix degradation were significantly regulated, including downregulation of the collagen crosslinking enzymes LOXL3 and P3H3 and upregulation of the collagen catabolizing MMP2 and MMP13, while all significantly regulated ATPase proton pumps were upregulated. The analysis of post-translational modifications of proline oxidation on the identified proteins showed significant repression of several specific modifications associated with different collagen species (**h**). *n* = 5 per group

An RNA-seq analysis of WT male cortical bone showed great repression of several LOX and P3H family members with age (Fig. 6a, b). Relative to young bone, transcript levels for *Lox*, *Loxl1*, and *Loxl2* displayed sustained repression up to 2 ½ years, whereas *Loxl3* and *Loxl4* levels were decreased at the 1-year timepoint. Of the P3H enzymes responsible for proline oxidation that enables tropocollagen helix formation, only *P3H2* did not show significant and prolonged age-related transcriptional repression. Using RT‒qPCR, we investigated whether the expression of candidate collagen crosslinking enzymes was sensitive to changes in osteocytic TGFβ signaling with age. The *Loxl3* mRNA levels were repressed at 4 and 15 months in male *TβRII*^*ocy−/−*^ bone compared to age-matched Cre-negative controls (Fig. 6c), whereas females displayed only an age-related repression of *Loxl3* expression in Cre-negative control mice (Fig. 6d), similar to the regulation of Loxl2 and Periostin (Fig. S5).

Given that bone matrix turnover clears accumulated crosslinks, we hypothesized that nonenzymatic crosslinks, including AGEs, would increase with age and in young *TβRII*^*ocy−/−*^ bone.^26,34^ For both males and females, AGE levels, normalized to collagen content, were indistinguishable between control and *TβRII*^*ocy−/−*^ bones at 4 months (Fig. 6e, f). By 12 months, AGE levels in both male and female control bones, as expected^40^ increased. Interestingly, both male and female *TβRII*^*ocy−/−*^ bone showed an additional level of AGE accumulation compared to their age-matched genotype controls at 12 months, such that AGE levels were highest in aged *TβRII*^*ocy−/−*^ bones.

Fourier transform infrared spectroscopy (FTIR) can detect bulk changes to chemical structures that result from altered bone composition, collagen maturity, or enzymatic activity. FTIR of young male *TβRII*^*ocy−/−*^ bone showed a significant reduction in the amide I peak area compared to that in relation to age-match Cre-negative controls, representative of reduced enzymatic collagen crosslinking (Fig. S6a, b). This result is consistent with the dysregulation of collagen composition and the decline in maturity of male *TβRII*^*ocy−/−*^ bone.^60–62^ As in other outcomes, young female bones did not show osteocytic TGFβ-dependent differences in FTIR compositional analysis (Fig. S6c, d).

A proteomic analysis of young Cre-negative control bone and *TβRII*^*ocy−/−*^ bone provided critical insight into the osteocytic mechanisms that regulate collagen PTMs and bone quality. Mass spectrometric analysis identified 2015 quantifiable proteins with UniProtKB-TrEMBL IDs, each with at least 2 unique peptides associated with the full protein. Of these 2015 quantified total proteins, the relative abundance of 186 proteins was significantly altered with genotype (Fig. 6g). Several critical bone matrix regulatory enzymes, including MMP2, MMP13, ACP, and carbonic anhydrases 2 (CA2) and 3 (CA3), were differentially expressed in *TβRII*^*ocy−/−*^ bone. Some, but not all, mirrored transcriptional changes observed with aging, including ACP5, LOXL3, and P3H3, were repressed in the aging bone transcriptome and in the young *TβRII*^*ocy−/−*^ bone proteome. Accordingly, post-translational proline oxidation sites of collagen peptides, the product of P3H activity, were significantly decreased (Fig. 6h) (Table 3). ADAMTLS2, a secreted glycoprotein that is regulated by TGFβ signaling, whose mutations are associated with connective tissue dysplasia, was also downregulated.^63,64^ Finally, all significantly altered ATPase subunits, including ATP6v0d2 and others implicated in pericellular acidification during lactation-induced PLR, were upregulated in the *TβRII*^*ocy−/−*^ proteome, as in the aging male bone transcriptome presented in this study.

**Table 3:** Posttranslational modifications with the *TβRII*^*ocy−/−*^ proteome

UniProt ID	Protein of Origin	Description	Peptide Sequence	Oxidation Site	Log_2_ (Normalized FC)	*P* value
P11087	Col1a1	Collagen alpha-1(I) chain	1	P-1169, P-1171, P-1172, P-1175, P-1181, P-1194	−0.94	3.24E-03
Q01149	Col1a2	Collagen alpha-2(I) chain	1	P-1098, P-1101, P1104, P1107	−4.97	1.97E-04
Q01149	Col1a2	Collagen alpha-2(I) chain	2	P-846, P-854, P-864, P-876, P-882	−3.1	2.93E-03
Q01149	Col1a2	Collagen alpha-2(I) chain	3	P-1026, P-1038, P-1050, P-1052	−3.87	4.49E-03
Q01149	Col1a2	Collagen alpha-2(I) chain	4	P-987	−2.15	8.59E-03
P08122	Col4a2	Collagen alpha-2(IV) chain	1	P-1416, P-1420, P-1423	−2.81	2.73E-03
O88207	Col5a1	Collagen alpha-1(V) chain	1	P-537	−1.8	1.44E-03
O88207	Col5a1*	Collagen alpha-1(V) chain	1	P-537	−1.32	7.34E-03
Q02788	Col6a2	Collagen alpha-2(VI) chain	1	P-719	−2.35	6.31E-04
Q61245	Col11a1	Collagen alpha-1(XI) chain	1	P-505	−1.52	2.16E-03
Q64739	Col11a2	Collagen alpha-2(XI	1	P-465	−1.36	8.06E-03
Q61702	Itih1	Inter-alpha-trypsin inhibitor heavy chain H1	1	P-321	−3.36	5.96E-03
P19221	F2	Prothrombin	1	P-517	−2.99	6.02E-03
P19221	F2	Prothrombin	2	P-373	−2.78	7.03E-03

Significant differences in posttranslational modifications between the proteome of young male control and *TβRII*^*ocy−/−*^ bone are shown, including a downregulation of proline oxidation at multiple sites of collagen subtypes and other proteins. * = methionine oxidation, an allowable modification in the search; indicates a different precursor from the previous peptide in the row above

Few proteins or PTMs showed a differential abundance in the proteomic analysis of young female *TβRII*^*ocy−/−*^ bone compared to the controls (Fig. S7A, B), which was similar to the lack of detectable differences in females for other outcome measures. Interestingly, significant changes in the abundance of 11 out of 1 145 detected proteins included TGFβ2 ligand, which was slightly upregulated in female *TβRII*^*ocy−/−*^ bone, as were three specific collagen proline modifications. Increased levels of the TGFβ2 ligand were also detected by ELISA in female *TβRII*^*ocy−/−*^ bone relative to the controls, but these differences were detectable only at 12 and 15 months of age (Table S5), reflecting the sensitivity of mass spectrometry analysis over traditional ELISA techniques. Together, this material and molecular examination of the role of osteocytic TGFβ signaling in bone aging identifies impaired collagen strain and the coordinated deregulation of collagen posttranslational modifications that may contribute to the age-related decline in bone quality.

## Discussion

**Fig. 7:**
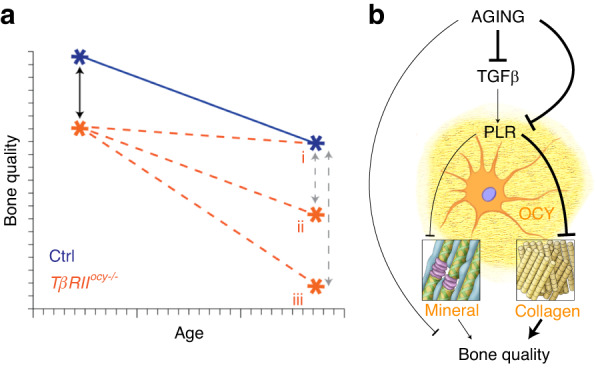
Schematic for the age-related control of bone quality through TGFβ signaling. By aging both male and female *TβRII*^*ocy−/−*^ bone alongside Cre-negative controls, we examined the combinatorial effects of age, osteocytic TGFβ signaling, and PLR in the control of bone quality (**a**). The aging trajectories of male and female *TβRII*^*ocy−/−*^ bone discriminate among the many age-dependent changes to identify those that are i) TGFβ dependent, ii) TGFβ independent, or iii) more susceptible to impaired osteocytic TGFβ signaling with age. These relationships further revealed a set of coregulated molecular and material mechanisms (**b**) by which age, and potentially other pathological or therapeutic factors, can act on osteocytes to calibrate bone quality by modulating the organic bone extracellular matrix independently of its mineralized constituents

Here, we demonstrate an osteocyte-intrinsic role of TGFβ signaling in the age-related decline in bone quality (Fig. 7a). By dissecting the effects of skeletal aging downstream from osteocytic TGFβ signaling, we identify outcomes that are coregulated in an age- and sex-specific manner, which provide fundamental insight into the molecular and material mechanisms that determine bone quality. Specifically, we find that the transcriptional evidence of TGFβ signaling in bone declines with age and that the effect of osteocytic TGFβ deficiency generates a premature bone aging phenotype in males, but not females, by altering collagen crosslinking and integrity. The age-related degeneration of the osteocyte LCN in controls, decline in collagen material properties, and increase in bone stiffness result from the loss of osteocytic TGFβ signaling. On the other hand, the effects of age on bone mass, mineral density, and material behavior occur independently of osteocytic TGFβ signaling. Additionally, we found that the maintenance of bone quality in young females is independent of osteocytic TGFβ signaling, an effect that diminishes with age. Thus, outcomes related to osteocyte cellular behaviors, such as matrix remodeling through the expression of collagen-modifying enzymes, correlate with material and tissue outcomes, including LCN integrity, collagen posttranslational modification, and collagen strain capacity, and are independent of age, sex, or genotype. Accordingly, we assert that osteocytes are key mediators in the age-related decline in bone quality. The decline in bone quality with age arises from a TGFβ-dependent loss of osteocyte function, which compromises the homeostatic maintenance of the bone pericellular matrix and the material performance of collagen in resisting fracture.

We find decreased levels of TGFβ in aged bone. The bone matrix serves as the body’s repository of latent TGFβ ligand, which is released and activated during bone resorption.^23^ Thus, age-related changes in bone cell behavior may impact the local or systemic levels of TGFβ. Indeed, increases in systemic TGFβ contribute to age-related cellular senescence, inflammation, neurodegenerative disorders, and cancer, among other conditions.^65^ In bone, however, we find that the TGFβ ligand levels and transcriptional markers of TGFβ signaling decline with age. These findings suggest that increased bone resorption with age may sustain circulatory TGFβ levels while simultaneously depriving osteocytes of a signal needed to support bone homeostasis. The altered aging and fragility phenotype in *TβRII*^*ocy−/−*^ bone^26^ indicates that the reduced TGFβ signaling in aged bone may be sufficient to elicit skeletal aging.

By examining the *TβRII*^*ocy−/−*^ bone phenotype over time, we demonstrate a relationship between the age-related loss of TGFβ signaling and skeletal fragility. Age-related bone fragility is associated with a multitude of changes to cellular function, material composition and properties, microscale geometry, and other factors that ultimately impact whole-bone toughness and strength. We distinguish the TGFβ-dependent (Fig. 7bi) and TGFβ-independent (Fig. 7bii) parameters of aging bone. Factors regulated in an osteocytic TGFβ-dependent fashion include the integrity of the LCN, the material behavior of the collagen matrix, and the postyield macromechanical behavior. For example, the LCN of *TβRII*^*ocy−/−*^ bone is indistinguishable between 4 and 15 months in males, whereas Cre-negative control males show progressive loss of LCN integrity with age. For LCN integrity and other osteocytic TGFβ-dependent parameters, osteocytic deficiency in TGFβ signaling is necessary and sufficient to generate the aged phenotype (Fig. 7bi). While TGFβ impacts both mineral density and material performance, age affects bone mineral in a TGFβ-independent manner (Fig. 7bii). Several other macromechanical (i.e., stiffness and strength by ultimate force, Fig. 3b, c) and structural (i.e., MOI, porosity, Fig. 2d, e) outcomes demonstrate a compounding effect of lost osteocytic TGFβ signaling and age (Fig. 7biii). Importantly, osteocyte LCN, collagen material behavior, and macroscale postyield displacement are found to be coregulated in the same manner across age, genotype, and sex, indicating that these three parameters are subject to coordinated biological control through osteocytes. Thus, tailoring osteocyte-targeted therapies to maintain the integrity of the osteocyte LCN and the collagen matrix may be a feasible way to prevent age-related fragility fractures, even in individuals with clinically normal bone mass.

The sensitivity of these multimodal outcome measures, together with genetic mouse models, affords the chance to interrogate the molecular and material mechanisms by which osteocytes control bone material quality. The proteomic and SAXS analyses of collagen crosslinking and material behavior proved especially powerful. Relative to that of young Cre-negative control male mice, young male *TβRII*^*ocy−/−*^ bone and aged Cre-negative control bone are unable to stretch collagen d-spacing, driving down the collagen-to-tissue strain slope. Enzymatic and nonenzymatic collagen crosslinking dictates this behavior. Therefore, the repression of several lysyl and prolyl hydroxylase collagen crosslinking enzymes in both aged and *TβRII*^*ocy−/−*^ bone can be tied to these changes to bone material performance. FTIR and proteomics further confirmed the consequence of repressed collagen-modifying enzymes by identifying a loss of collagen PTMs in young male *TβRII*^*ocy−/−*^ bone relative to Cre-negative controls. By incorporating the analysis of nonenzymatic AGEs, we can further discern the effects of the time- or genotype-dependent loss of TGFβ signaling on collagen material to demonstrate age-related stiffening on the nanoscale. In a manner parallel to SAXS, WAXD scans demonstrate a loss in mineral crystal-associated strains in *TβRII*^*ocy−/−*^ bones but no age-dependent change in either genotype. Additional research is needed to determine whether the upregulation in young *TβRII*^*ocy−/−*^ male bone of ATPase ion pumps and other pH-regulatory enzymes, as detected by RNA- seq and mass spectrometry (Fig. 5a, Fig. 6g), are the molecular causes of the genotype-dependent differences in mineral strain. Collectively, these findings demonstrate an age-related material mechanism by which impaired TGFβ signaling in osteocytes negatively affect the bone matrix, specifically collagen posttranslational modifications, that in turn affect overall bone material quality, contributing to bone fragility independently of bone mass.

Apart from the molecular regulation of collagen posttranslational modifications, other osteocyte functions change upon the loss of TGFβ signaling, PLR, and bone matrix synthesis. Each of these factors has the potential to impact bone quality. First, consistent with prior results,^12,44^ several markers of osteocyte senescence increase with age, as well as with the suppression of osteocytic TGFβ signaling (Fig. 1a–d, Fig. 6o). The induction of a senescence-associated secretory phenotype (SASP)^66^ has the potential to affect bone quality in myriad ways, including altered protease expression or the mitochondrial dysregulation of reactive oxygen species.^67,68^ However, the extent to which increased osteocytic senescence in aging is TGFβ-dependent remains to be determined. Second, we and others previously showed the necessity of enzymes implicated in osteocyte PLR in the maintenance of the LCN, collagen organization, and bone quality.^26,27,29,31,69,70^ These enzymes include the proteases MMP2, 13, 14 and cathepsin K, which could alter the material behavior of collagen, and proteins controlling acidification, such as carbonic anhydrase 2 and ATPases, which could alter the material behavior of bone mineral. Therefore, we anticipated that one or more of these enzymes would show coordinated control by age and osteocytic TGFβ signaling. Although many of these enzymes showed regulation by age and suppressed osteocytic TGFβ signaling in a similar manner, none of the candidate genes we examined by qRT‒PCR showed evidence of dual regulation by age and TGFβ. However, proteomic analysis did indicate significant repression of LOXL2 and P3H3 protein levels in young male *TβRII*^*ocy−/−*^ bone, indicating deregulated enzymatic control of collagen, similar to aged WT bone. Additionally, many other isoforms of these enzymes, including LOX, LOXL2, and LOXL4, were detected by proteomic approaches in the same aliquot, improving upon qPCR by the unbiased detection of many proteins and posttranslational modifications at once. These enzymes were similarly downregulated with a significance of q < 0.05, but fold changes were approximately 20% instead of the ≥50% significance threshold in this analysis (Table S3). Other collagen regulatory enzymes, including P3H1, P3H4, PLOD1, and PLOD2, were also detected in the proteomic analysis, and although these did not show regulatory effects, their presence in the dataset demonstrates the power and specificity of our proteomics approach in investigating molecular mechanisms that can regulate the bone ECM. Among many ways that PLR suppression may compromise bone quality is the accumulation of spontaneously generated nonenzymatic crosslinks due to insufficient peri-osteocytic matrix turnover. Indeed, nonenzymatic crosslinks accumulate with age, ultimately exceeding their enzymatic counterparts by 5-10-fold over time,^40^^,41^ which negatively impacts collagen ductility, sacrificial bonding, and interfibril sliding during deformation.^71^ Third, age and TGFβ-dependent changes in the expression and modification of noncollagenous matrix proteins (i.e., OCN, OPN, BSP)^72,73^ could impact collagen material behavior, even independently of mineral, which is consistent with the results of the SAXS studies here. Although transcriptional analysis did not detect age-related specific regulation of these proteins, the possibility remains that noncollagenous proteins (NCP) and PLR enzymes are regulated at the posttranscriptional level. Additional research will be needed to dissect the causal role of NCPs in the age-related loss of bone quality.

As we previously reported, the role of osteocytic TGFβ signaling is sexually dimorphic. Osteocytic TβRII signaling is needed for lactation-induced PLR and is essential for males, but dispensible for females in basal conditions, in the maintenance of the LCN and bone quality. The mechanisms by which young female bones compensate for the loss of osteocytic TβRII remain unknown but could conceivably involve the utilization of other TGFβ family receptors^20,25,74^ or other mechanisms. For instance, female *TβRII*^*ocy−/−*^ bone showed a small upregulation of TGFβ2 ligand in mass spectrometry analysis (Fig. S4A), perhaps compensating for the loss of the TGFβ receptor. Although the current study tested the hypothesis that the dispensability of osteocytic TGFβ signaling in young female bone would be lost with age, the results were inconclusive. Aging female bone showed canalicular loss and increased network tortuosity independent of genotype. Highly sensitive SAXS detected a decrease in the collagen-to-tissue strain slope in aged female bones that appears to be TGFβ dependent, as it is in males. However, we concluded that in young males, this loss is associated with a loss in collagen fibril straining accompanied by fibril slipping in the absence of both enzymatic and nonenzymatic crosslinking, while previous SAXS studies^11,59^ showed that this change in slope was associated with tissue stiffening and increased crosslinking. This interpretation is consistent with the observations of aged control females in our study, in which AGEs increased with age; however, aged *TβRII*^*ocy−/−*^ females retained young-like behavior on the nanoscale despite further increased AGE content. Indeed, osteocytic TGFβ signaling is certainly not the only factor responsible for changes in collagen behavior or losses of bone quality with age. For example, the young *TβRII*^*ocy−/−*^ female bone phenotype suggests that a decline in bone TGFβ levels with age may be necessary, but not sufficient, to generate the aged-bone phenotype. Thus, additional studies are needed to determine the basis of the age-dependent changes in female bone collagen strain.

The tissue-level outcomes of μCT and three-point bending in aged female bone add further complexity. Given the age-related decreases in female bone TGFβ ligand levels, LCN integrity, and postyield displacement, these findings suggest that female bones acquire a need for osteocytic TGFβ signaling with age. Longer endpoints or more sensitive outcomes will be needed to understand the role of osteocytic TGFβ signaling in older female bone more fully, including elucidating the age-related relationship of collagen quality and the role of enzymatic crosslinking.

A comprehensive analysis of the molecular and material mechanisms controlling bone quality in young and old, male and female, *TβRII*^*ocy−/−*^ and Cre-negative control bone yielded fundamental insight into the biological mechanisms controlling bone quality. While these factors implicate the control of collagen posttranslational modifications and material behavior by osteocytes, many other mechanisms are undoubtedly involved, including changes in bone geometry, porosity, and other parameters. Additionally, the use of constitutively active DMP1-Cre to remove TGFBR2 can implicate earlier stages of the osteogenic line, including osteoid-producing osteoblasts. The multiple time points in our study revealed the dynamic control of proteases and other collagen-modifying enzymes, including MMPs, Ctsk, Loxls, and P3Hs, in both WT-type mice and the *TβRII*^*ocy−/−*^ line. The expression of Cre recombinase from the 10 kb DMP1-Cre may also cause recombination of floxed alleles in other cell types, so the effects may arise from cells other than osteocytes.^75^ Thus, the osteoblast-intrinsic contributions to collagen posttranslational modifications and the extent to which this relates to developmental vs. homeostatic functions for these enzymes remain unexplored. However, considering our prior works, it is unlikely that developmental or osteoblastic mechanisms alone are sufficient to account for the changes in the properties of the bone ECM. For example, in 2-month-old mouse cortical bone, maximal levels of MMP13 were detected in the most mature mid-cortical diaphyseal bone, with lower expression in the more osteoid-rich endosteal and periosteal surfaces.^31,76^ In addition, the consequences of MMMP13 deletion were most profound in this more mature bone. Future studies that examine the full transcriptomic profile of aged bone, especially including late time points (2 yr, 2 ½ yr) in female WT mice to match the aged males, as well as in older female *TβRII*^*ocy−/−*^ bone, will also be important next steps to extend upon previously published osteocyte transcriptomes from young and middle-aged WT female bone.^77^ Additionally, newly advancing spatial techniques that can elucidate the periosteal to endosteal gradients of enzymatic activity within bone would greatly enhance our knowledge of the contributions of different cell types to the regulation of the ECM. This may also elucidate phenotypes unexplored here, such as the role of increased cortical porosity in the midshaft presented by the *TβRII*^*ocy−/−*^ line.

The identification of the relationship between TGFβ signaling and age in bone elucidates a mechanism that is responsible for the biological control of collagen material quality. Furthermore, we found that this mechanism contributes to the age-related decline in bone quality, providing critical insight into a currently unmet clinical need. Beyond age- and genotype-dependent differences in collagen-modifying enzyme expression and posttranslational modifications (Fig. 6), many questions remain about the cellular mechanisms by which osteocytes participate in the age-related decline in bone matrix material properties. The current work can inform future studies, for example, using GFP-collagen mice or easily manipulatable click chemistries,^45,78^ to discover these mechanisms by focusing attention on collagen rather than minerals and on enzymatic rather than nonenzymatic crosslinking. By identifying osteocytes as active players in bone aging, current research will motivate the future analyses of other osteocytic mechanisms that are affected by age, such as cellular senescence and proteolysis. These mechanisms present prime opportunities to defend against nonosteoporotic fracture resulting from the loss of bone quality in aging individuals.

## Materials and Methods

Complete details are contained in the Supplemental Extended Methods.

### Murine studies

Male C57BL/6 mice (WT, wild type) aged 2 months (*n* = 3), 1 year, 2 years, and 2 ½ years (30 months) (*n* = 4) were received from the Buck Institute for Research on Aging. To interrogate the role of osteocyte-intrinsic TGFβ signaling, mice possessing *loxP* sites flanking exon 4 of the Tgfbr2 gene were crossed with hemizygous −9.6 kb-*DMP1-Cre*^*+/−*^ mice.^79–81^ In *DMP1-Cre*^*+/−*^;*TβRII*^*flfl*^ (*TβRII*^*ocy−/−*^) mice, TGFβ receptor II was selectively ablated in osteocytes. *DMP1-Cre*^*−/−*^;*TβRII*^*fl/fl*^ (Cre-negative Control) littermates were used as controls. All mouse genotypes were confirmed by PCR genotyping.^26^ Male and female *TβRII*^*ocy−/−*^ and Ctrl mice were aged naturally to 4 months (16 weeks), 6 months (24 weeks), 12 months (48 weeks), and 15 months (60 weeks) (*n* = 7–12 per group). All animal procedures were approved by the Institutional Animal Care and Use Committee of the University of California San Francisco and the Buck Institute for Research on Aging.

### RNA isolation and analysis of gene expression

The soft tissue and periosteum were removed from mouse humeri; the ends were dissected at the metaphysis; and the marrow removed via centrifugation. The diaphyseal cortical bone was snap-frozen in liquid nitrogen and then added to tubes containing ice-chilled QIazol (Invitrogen). Bone samples in solution were mechanically homogenized with a rotor-stator homogenizer (GLH, Omni) and then stored at −80 °C.^26,32,82^ mRNA was purified using the miRNeasy Mini Kit (Qiagen), following the manufacturer’s instructions with on-column DNase (Qiagen) digestion to remove genomic DNA, as previously described.^83^ The RNA concentration was quantified with a NanoDrop spectrophotometer. The RNA was stored at −80 °C.

RNA sequencing of the aged WT C57BL/6 mice was completed as described on the Illumina HiSeq 4000 at the UCSF Functional Genomics Core.^83^ Briefly, single-end reads were aligned to the Ensembl mouse reference genome using STAR 2.5.2b aligner^84^. The DESeq2 package in R Statistical Computing Environment^85^ was used for differential expression analysis and hierarchical clustering. The read count fold change for individual gene comparisons was normalized with reference to the average read count of the young (2-month) WT mice. Statistical comparisons were performed with the Bonferroni correction and Student’s T test for six multiple comparisons over time among the four age groups.

For RT‒qPCR, RNA from each sample (*n* = 5-6 per group) was reverse-transcribed to a final uniform concentration using the iScript cDNA synthesis kit (Bio-Rad). RT‒qPCR of cDNA utilized iQ SYBR Green Supermix (Bio-Rad) with 18 s as the housekeeping gene for the comparison of target genes *Loxl2, Loxl3*, and *Postn* (Table S1) on a Bio-Rad Thermocycler (Hercules, CA). Gene expression was quantified by the ΔΔCt method.

### TGFβ ELISA

Protein from bone lysates (12-15 per age and sex) was harvested as previously described^86^ using Bead Mill Tubes (Thermo Fisher Scientific, Waltham, MA) containing radioimmunoprecipitation (RIPA) buffer with eComplete Mini protease inhibitor (Roche, Basil Switzerland). and homogenized using a benchtop bead mill homogenizer (Omni International, Kennesaw, GA) at 4 °C. Serum was collected from blood as previously described.^27^ Protein concentrations were quantified using the Pierce Coomassie (Bradford) Assay Kit (Thermo Fisher Scientific, Waltham, MA). Total levels of TGFβ ligand were quantified in hydrolyzed lysates by using the Bio-Plex Pro TGF-β Assay Kit (Bio-Rad, Hercules, CA) on a Bio-Plex 200 (Bio-Rad, Hercules, CA). TGFβ ligand levels were normalized to each sample’s original total protein concentration.

### Histological analysis

Femurs (*n* = 5–6 per group) were stripped of periosteum and connective tissue, fixed in 10% neutral buffered formalin (NBF), decalcified in EDTA (pH 7.3-4) and prepared for cryosectioning as previously described.^34^^,82,87^Axial sections of the femurs were stained with the lipophilic dye 1,1′-Dioctadecyl-3,3,3′,3′-Tetramethylindocarbocyanine Perchlorate (DiI, ThermoFisher) dissolved in 50% PBS/DMSO and Phalloidin-conjugated Alexa Fluor 488 (Invitrogen) for the labeling of osteocyte membranes and cytoskeleton, respectively.^80^^,82^ Sections for 3D imaging were taken from the midpoint of the diaphyseal femur halfway between the growth plates. Given the ages and sexes analyzed, the absolute distance changed per animal, so this method was used to obtain consistent regions of interest. Each sample was sectioned at a size of 50 μm, moving from the distal end of the bisected proximal femur midshaft toward the femoral head. Each digitally analyzed region was 35 μm in thickness and excluded ~7.5 µm on either edge of the image stack to avoid imaging artifacts. Histological TUNEL assays were completed on 8-10 μm cryo-sections using the Fluorescein In Situ Cell Death Detection Kit from Roche (Basel, Switzerland). Cell death was measured as the percentage of identified cell nuclei contained with DAPI and fluorescein against the total number of DAPI-positive nuclei. Fluorescent confocal imaging was completed using a Lecia DMi8 (Leica Microsystems, Wetzlar, Germany) inverted confocal microscope operating LAS X software, while brightfield imaging was completed with a Nikon Eclipse E800 microscope (Tokyo, Japan). 3D structural analysis of the osteocyte LCN was completed as described.^82^

### μCT

Unfixed femora (*n* = 8–12 per group) were scanned using a Scanco μCT50 specimen scanner (Scanco Medical, Wangen-Brüttisellen, Switzerland) at room temperature in Hanks Balanced Sodium Salts (HBSS, Thermo Fisher Scientific, Waltham, MA). 3D measurements of cortical and trabecular parameters were obtained using Scanco analysis methods as described,^88^ while measurements of cortical porosity were collected through the manual segmentation and quantification of Scanco-generated DICOM files utilizing Dragonfly (Object Research Systems, Montreal, Canada).

### Mechanical testing

Whole hydrated femurs (*n* = 8–12 per group) were loaded to failure in three-point bending in the direction of primary physiological bending (posterior compression) using a Bose Electroforce 3200 test frame as previously described.^76^ After testing, fracture surface cross-sections were imaged by scanning electron microscopy on a Sigma 500 VP FE-SEM (Zeiss). The endosteal and periosteal cross-sectional diameter and thickness were measured with ImageJ and used to calculate the moment of inertia, assuming an elliptical cross-section for the calculation of material properties from structural parameters.^89^

### Synchrotron in situ tensile testing

Collagen fibril and hydroxyapatite mineral crystal strains were recorded during uniaxial tension testing of isolated, hydrated ulnae using synchrotron small-angle X-ray scattering (SAXS) and wide-angle X-ray diffraction (WAXD) at beamline 7.3.3 at the Advanced Light Source (LBNL, Berkeley, CA).^90^ This strategy permits the real-time measurement of strain in the collagen or mineral phases of bone along with simultaneous measurement of bulk tissue strain, as captured by a CCD camera. Each epiphysis of the ulnae was embedded in resin to create a “dumbbell” shape around the end of the bones for clamping and applying tensile strain without crushing the bone. In situ tensile tests (*n* = 8–12 per group) were performed with a TST350 Tensile Testing Stage (Linkam Scientific Inc.) on hydrated samples at RT as previously described.^91^ The mid-diaphysis of hydrated ulnae was exposed to a perpendicular X-ray beam with a spot size of 750 μm × 250 μm and an energy of 10 keV for 0.5 s every 5 s until failure or until the total radiation dose reached 30 kGy to limit the effects of radiation exposure on the material performance of the bone.^91^ Samples that slipped from the clamps, were removed from the resin prior to fracture, or showed a nondiaphyseal fracture were excluded from final analysis for a final *n* of 5–8 per group. X-ray scattering data were collected using a Pilatus3 2 M detector (Dectris, Ltd.), and were calibrated by fitting the scattering from a silver behenate standard using the APS Nika macros for Igor Pro.^92^ The changes in collagen *d*-spacing and mineral crystal lattice spacing from the unloaded condition, shown as a shift in the location of the first-order Bragg scattering peak from the SAXS pattern and the (002) Bragg diffraction peak in the WAXD pattern, were used to calculate the collagen and mineral-associated strains, respectively. Material-specific strains were determined from changes in the *d-* or lattice spacings over time and were time-matched to their tissue-level strain at each point as determined from CCD images of the bone during deformation using a custom MATLAB and LabVIEW program.^11^

### Quantification of nonenzymatic collagen crosslinking

Decalcified, mid-diaphyseal femurs (*n* = 5 per group) were hydrolyzed in HCl (24 h, 110 °C), and the fluorescence of neutralized lysates (excitation 370 nm, emission 440 nm) was referenced to a quinine sulfate standard and then normalized to the collagen content calculated from the amount of hydroxyproline, as measured by a colorimetric assay with absorbance at 560 nm, to quantify the advanced glycation end-products (AGEs) within bone.^59^

### Fourier transform infrared spectroscopy

For select groups, the cortical diaphysis from femurs (*n* = 5–7 per group) was dissected, and marrow was removed via centrifugation. Samples were fixed overnight and dehydrated in an ethanol series. The remaining sample moisture was removed via desiccation in a vacuum chamber containing Dri-Rite (Chicago, IL) for 1 h. Dried cortical bone samples were homogenized by grinding with a polished mortar and pestle to obtain a fine powder. Fourier transform infrared (FTIR) spectra were collected in attenuated total reflection (ATR) mode on a PerkinElmer Spotlight 200i FT-IR Microscopy System (Waltham, MA) that was made available by the UCSF Core Center for Musculoskeletal Biology and Medicine (CCMBM). To account for the possibility of genotype-dependent changes in bone mineral densities, uniform amounts (10 mg) of powdered bone material, enough to cover the ATR crystal, were analyzed per sample. The spectra were background corrected, normalized to the mineral content at the peak of the phosphate signal (~1 010–1 030 cm^−1^),^61,62^ and ATR corrected via Spectrum software (PerkinElmer).^60^ Prior to normalization, the raw intensities of the phosphate peak were compared to ensure that a comparable amount of mineralized bone was analyzed per sample, and normalization to this feature would not skew results elsewhere in the collected spectra.^93–96^

### Protein extraction, mass spectrometric analysis, and data processing

Femurs (*n* = 5 per group) were stripped of the soft tissue and periosteum; the epiphyses were dissected; and the marrow was removed via centrifugation. Diaphyseal cortical femurs were demineralized overnight in HCl at 4 °C and then stored at −80 °C. The frozen bones were subsequently pulverized with the SPEX SamplePrep 1600 MiniG tissue homogenizer in polycarbonate tubes cooled with liquid nitrogen. Pulverized samples were transferred into a fresh Eppendorf tube with 800 µL of extraction buffer (6 mol/L guanidine hydrochloride GuHCl; 10 mmol/L Tris-HCl; 50 mmol/L EDTA) and incubated on a benchtop rotator at 4 °C for 72 h.^96–98^ The samples were then spun down to obtain pellets to collect the supernatant, which contained soluble protein that was buffer exchanged to remove GuHCl. Samples were washed by centrifugation in Amicon 3 kD Centrifugal Filters (Fisher Scientific, Hampton, New Hampshire) at 12 000 × *g* for 20 min and resuspended in 500 µL of 10 mmol/L Tris-HCl (pH 7) three times. Protein aliquots were resuspended in Tris-HCl and quantified using a bicinchoninic acid assay (BCA). The samples were tryptically digested using an established S-Trap mini spin column (Protifi, Farmingdale, NY) method. Processed samples were resuspended in 0.2% formic acid, and indexed Retention Time Standards (iRT, Biognosys, Schlieren, Switzerland) were spiked into each sample according to the manufacturer’s instructions.^99^ Reverse-phase HPLC‒MS/MS analyses were performed in data-independent acquisition (DIA) mode (Table S2) on a Dionex UltiMate 3000 system coupled online to an Orbitrap Eclipse Tribrid mass spectrometer (Thermo Fisher Scientific, San Jose, CA).^100–102^ Each biological replicate (*n* = 5 for *TβRII*^*ocy−/−*^ and *n* = 5 for Ctrl) was acquired independently, each with technical duplicates. The DIA data files were processed in Spectronaut (version 15.6.211220.50606, Biognosys) using directDIA for both the protein and peptide levels. The protein level quantification was based on the peak areas of extracted ion chromatograms (XICs) of 3–6 MS2 fragment ions with local normalization and *q* value sparse data filtering applied^102^ (Table S3). We also analyzed the data while accounting for posttranslational (PTM) hydroxyproline-containing peptides (Table S4). For additional details, see the Supplemental Methods.

### Statistical analysis and comparison

Prism v.9 (GraphPad, San Diego, CA) was used for all statistical comparisons except for RNA-seq of aged WT mice, TGFβ ELISA, and proteomic results. A range of statistical approaches were utilized based on the specific needs of the data and experimental design *(see* Supplemental Methods). In general, comparisons were performed within each sex, and independent statistical tests were used as appropriate. Two-way ANOVA comparisons used factors of age and genotype. Significance (*P*) values for all interaction terms and main effects of age and genotype can be found in Table S5. Simple pairwise effects were investigated in post hoc comparisons using Fisher’s least significance difference test with a priori Bonferroni-corrected alpha levels. This approach is noted in figures, as indicated by the captions. Not all possible pairwise comparisons are scientifically meaningful, and power was not sacrificed for excessive comparisons. *P* values considered ‘trending’ between *P* < 0.1 and Bonferroni corrected alphas are noted in the figures. ELISA analysis had additional group complexity (age, sex, genotype, tissue source, and ligand subtype), so full ensemble results were first analyzed with univariable and multilevel linear mixed models (Table S6) of the log of concentration to determine what factors most strongly predicted the ligand concentration. Afterward, pairwise comparisons were completed with the nonparametric Wilcoxon signed-rank test with significant alpha values Bonferroni corrected for the specific number of comparisons being completed.

Differential proteomics expression analyses for *TβRII*^*ocy−/−*^ vs. Cre-negative Ctrl proteomes were performed using a paired *t* test, and *P* values were corrected for multiple testing using the Story method.^103^ For the protein level, protein groups with at least two unique peptides, *q* value < 0.05, and absolute Log_2_(fold-change) > 0.58 were considered significantly altered (Table S3). To quantify the hydroxyproline-containing peptides, posttranslational modification (PTM) fold-change values were normalized by the corresponding protein fold-change values, and hydroxyproline-containing peptides with *P* value < 0.01 and absolute Log_2_(normalized fold-change) > 0.58 were considered to be significantly altered (Table S4). Volcano plots were generated using the ggplot2 package^104^ in R (version 4.0.5; RStudio, version 1.4.1106).

### Supplementary information

Supplemental Figures

Supplemental Extended Methods

Supplementary_Table_1_RT-qPCR_Primer_Sequences

Supplementary_Table_2_DIA_Isolation_Scheme

Supplementary_Table_3_Proteomics_Protein_Level_Analysis

Supplementary_Table_4_Proteomics_PTM_Hydroxyproline_Analysis

Supplementary_Table_5_Two_Way_ANOVA_Interaction_and_Main_effects

Supplementary_Table_6_ELISA_Stats_Results

## Data Availability

The mouse bone proteomic datasets have been uploaded to the Center for Computational Mass Spectrometry MassIVE repository at UCSD and can be accessed using the following link: https://massive.ucsd.edu/ProteoSAFe/dataset.jsp?task=5b52ca7cb69940398a6b32f02d9a1046. • MassIVE ID number: MSV000091488. The transcriptomic data presented here are publicly available at the NCBI under BioProject PRJNA695408.
