# Supplementary material for: Aging impairs the osteocytic regulation of collagen integrity and bone quality
Source: Bone Res. 2024 Feb 26;12:13. doi: 10.1038/s41413-023-00303-7 (PMC10897167; doi:10.1038/s41413-023-00303-7)

Supplemental Figure 1: Overlapping 95% Confidence Intervals for uCT parameters for 15 month of Cre-negative controls and 4 month old TβRII$^{ocy-/-}$ males indicating statistically significant similarity.

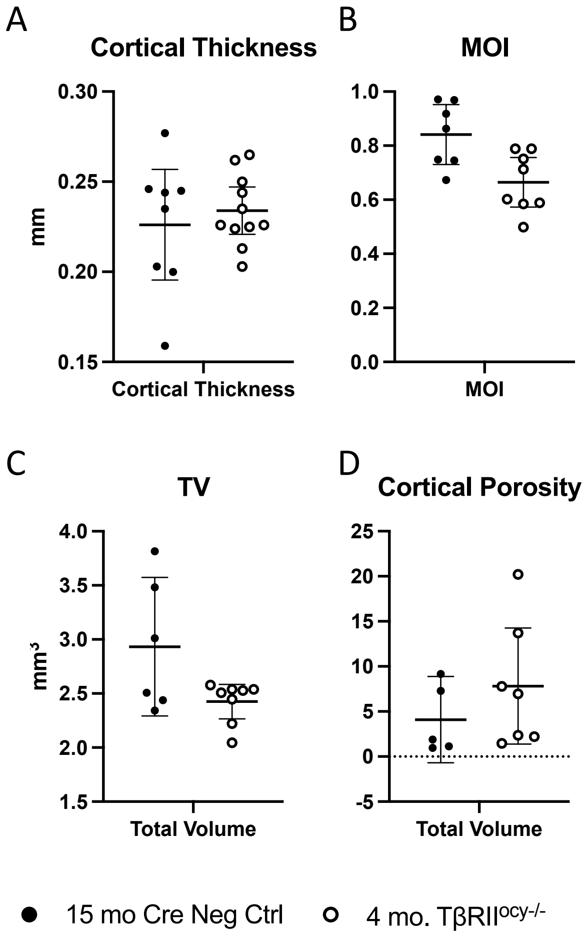

Supplemental Figure 2: Material Properties from 3-point Bending of Aging TβRII$^{ocy-/-}$ Bones

Bending modulus of male (A) and Female (B) Cre-negative Ctrls and age-matched TβRII$^{ocy-/-}$ littermates from stiffness values normalized by bone cross sectional area, and Yield (C,D) and Ultimate Stress (E,F) show no significant differences by genotype as analyzed Fischer's LSD test for multiple comparisons after two-way ANOVA. Male bending modulus does show a significant main effect of Age (p = 0.0308), regardless of genotype, while female bending modulus displays a simple main effect of Genotype (p = 0.0158). None of the yield or ultimate stress comparisons showed any significant simple main effects by Two-way ANOVA. There were no significant interactions.

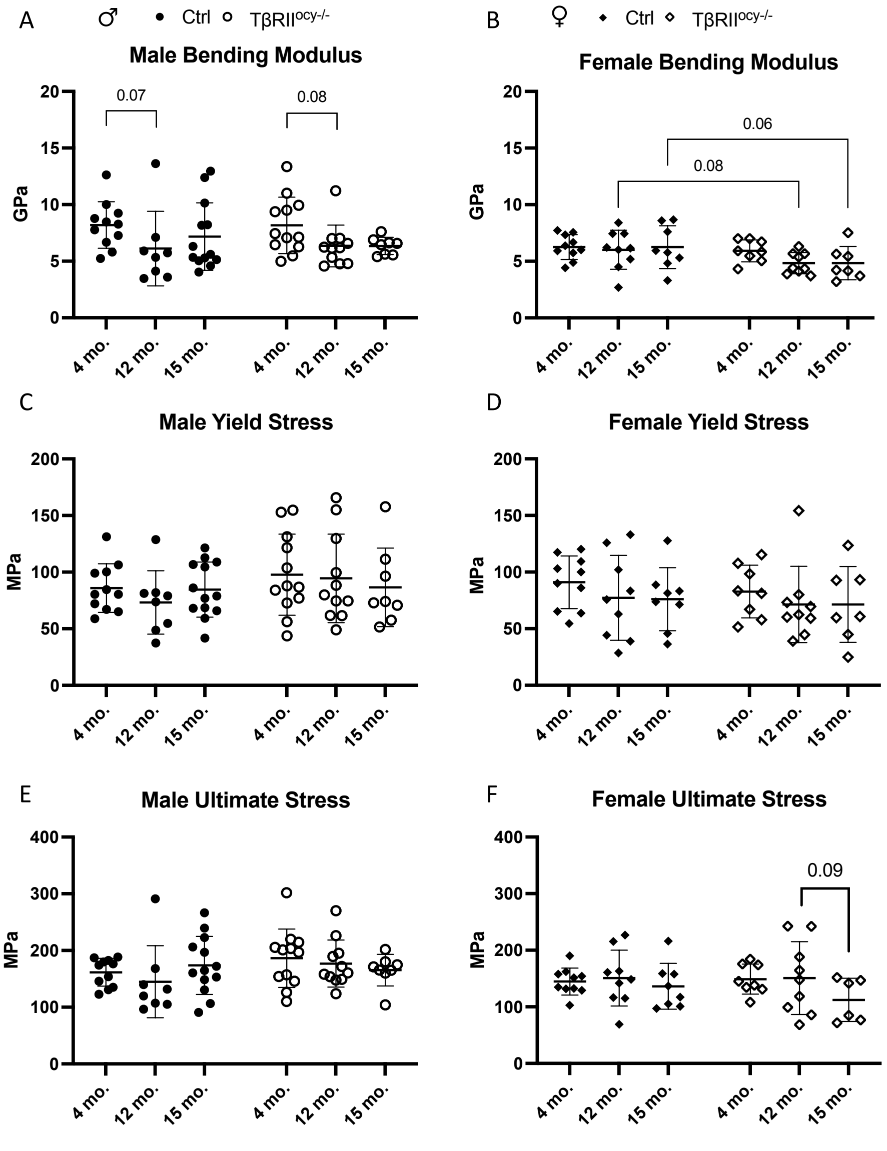

Supplemental Figure 3: Aging comparisons of nanoscale composite material strains within genotype.

Within-group comparisons of bone composite material performance with age reveal different effects of age between control and TβRII$^{ocy-/-}$ bone. The effects of age on collagen as seen through SAXS showed no significant changes in collagen strain between 4 and 15 months of age in male controls (A) while aging in TβRII$^{ocy-/-}$ males exhibited a time-dependent increase of collagen strain through an increase in the best-fit slope of tissue to collagen strain (B). Aging within female control bones revealed a dramatic decrease of collagen strain representing an ability to change collagen D-spacing under stress (C). This effect that was not seen in female TβRII$^{ocy-/-}$ bone (D). Evaluation of the effect of age on mineral strains through WAXD experiments showed no statistically significant age-related changes within genotype in either male or female bones (E-H). *$p<0.0125$ for 4-way Bonferroni correction in comparisons of regression slopes in an extra sum-of-squares F test. N=5-8 per group.

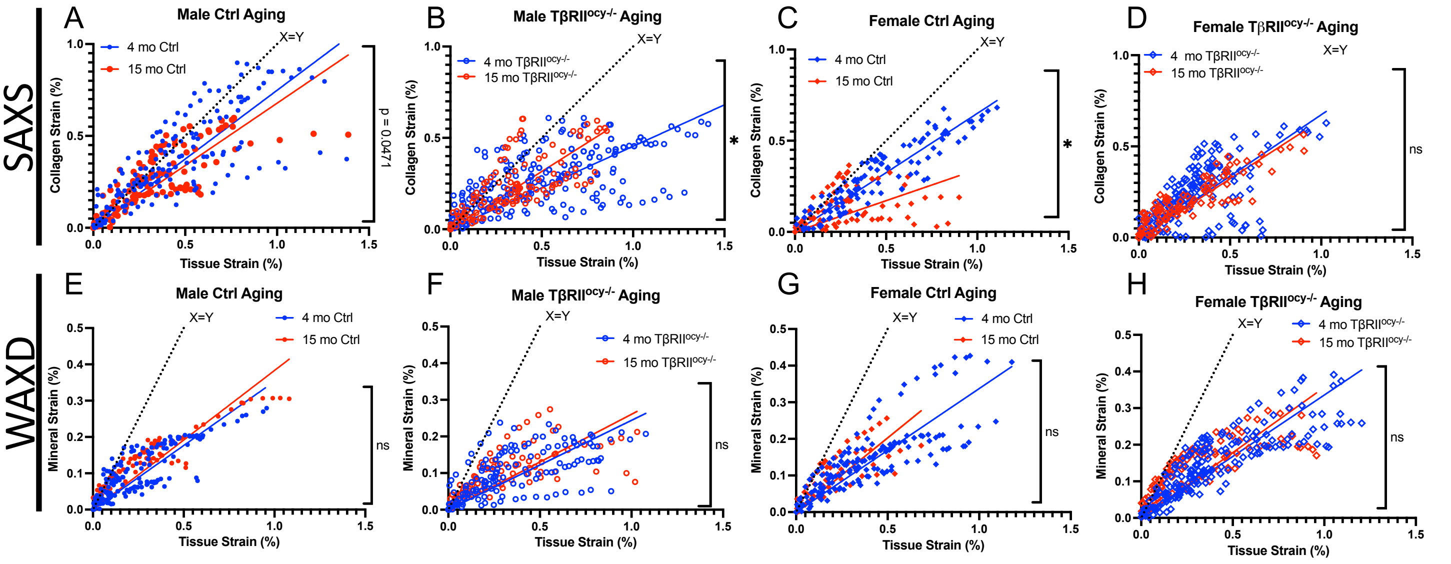

Supplemental Figure 4 Collagen order is lost with age in TβRII$^{ocy-/-}$ bone. Quantification of the Herman's Orientation factor (P2), or the Second Legendre coefficient, provides a measure of material order and shows loss of collagen order with both age and osteocytic deficiency in TGFβ signaling in males (A), whereas only female TβRII$^{ocy-/-}$ show lost order with age (B). Analysis of this parameter during tensile testing reveals collagen fibril motion in response to mechanical strain. Young male TβRII$^{ocy-/-}$bones do not change P2 (a fit slope nonsignificantly different from zero) during testing, indicating that collagen fibrils fail to align with strain. Collagen fibrils in control males, and young females of both genotypes, become more aligned with strain (C,D). Genotype-dependent differences in the ability of male bone to align collagen with strain are lost with age (E), whereas aged TβRII$^{ocy-/-}$ females show increased collagen alignment compared to controls (F).

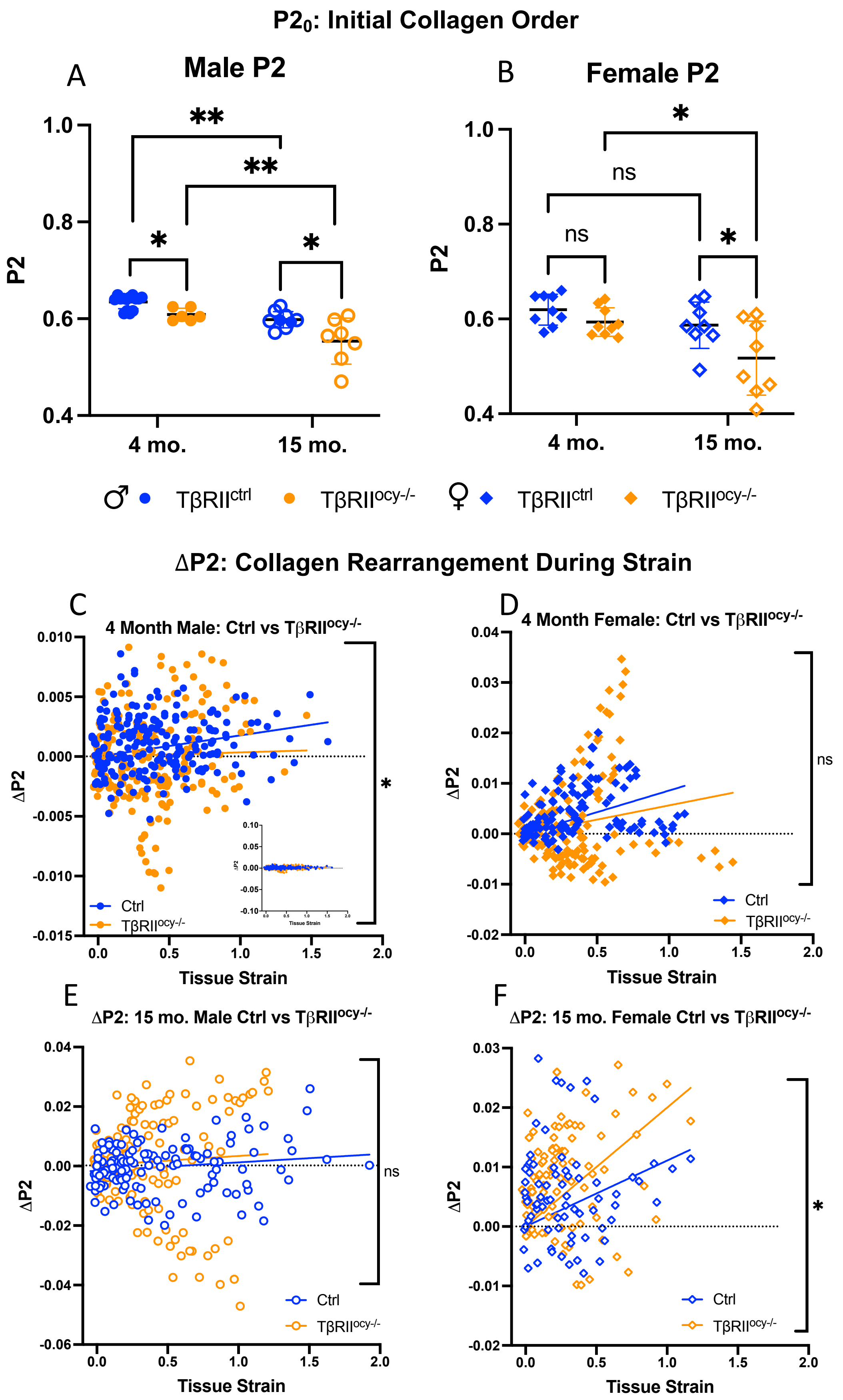
P2₀: Initial Collagen Order
A
Male P2
B
Female P2
P2
1.0
0.8
0.6
0.4
**
**
*
*
ns
ns
*
*
4 mo.
15 mo.
♂ TβRIIctrl TβRIIocy-/- ♀ TβRIIctrl TβRIIocy-/-
ΔP2: Collagen Rearrangement During Strain
C
4 Month Male: Ctrl vs TβRIIocy-/-
D
4 Month Female: Ctrl vs TβRIIocy-/-
E
ΔP2: 15 mo. Male Ctrl vs TβRIIocy-/-
F
ΔP2: 15 mo. Female Ctrl vs TβRIIocy-/-
ΔP2
Tissue Strain
Ctrl
TβRIIocy-/-
ns
*
0.0
0.5
1.0
1.5
2.0

Supplemental Figure 5: Additional RT-qPCR in TβRII$^{ocy-/-}$ bone. Expression levels of Loxl2 and Postn were analyzed from RNA of osteocyte enriched cortical bone from young (4 mo) and aged (15 mo) male and female TβRII$^{ocy-/-}$and controls. Males showed significant repression of Loxl2 from 4 to 15 months in TβRII$^{ocy-/-}$ bone, with no change in controls with age (A), while Periostin (Postn) showed marginal loss in TβRII$^{ocy-/-}$ bone with age (B). Females showed an age-dependent loss in expression levels of both Loxl2 and Postn within each genotype, but with no significant genotype-dependent differences at either age (C,D). *$p<0.0125$ (4-way Bonferroni correction after two-way ANOVA).

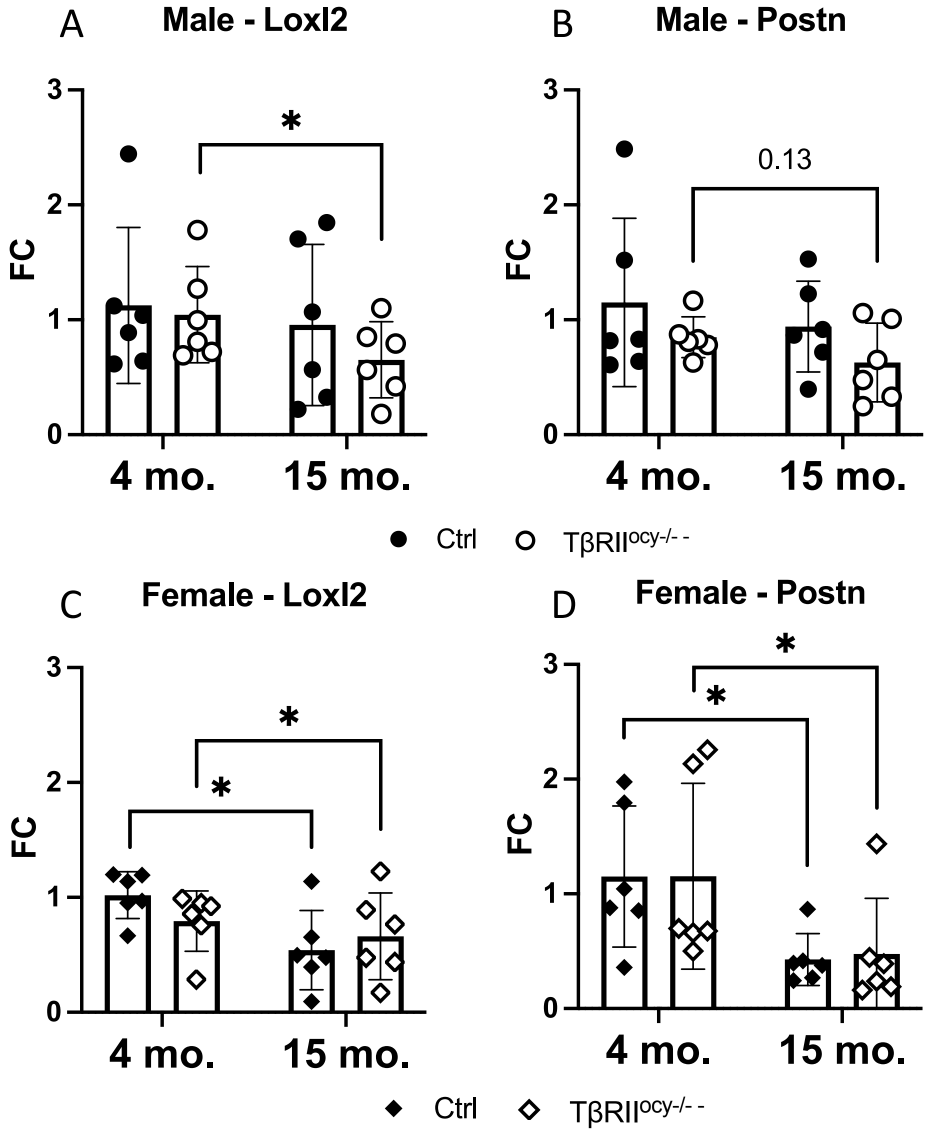

Supplemental Figure 6: FTIR analysis of TβRII$^{ocy-/-}$ bone: Fourier-transform Infrared Spectroscopy of 4-month male TβRII$^{ocy-/-}$ bone (A) showed repression of the Amide I peak (B) between 1590 and 1700 cm^{-1} while females (C) saw no noticeable differences between genotypes in this region (D). *$p<0.05$ in Student's T-test, N=5-7 per group.

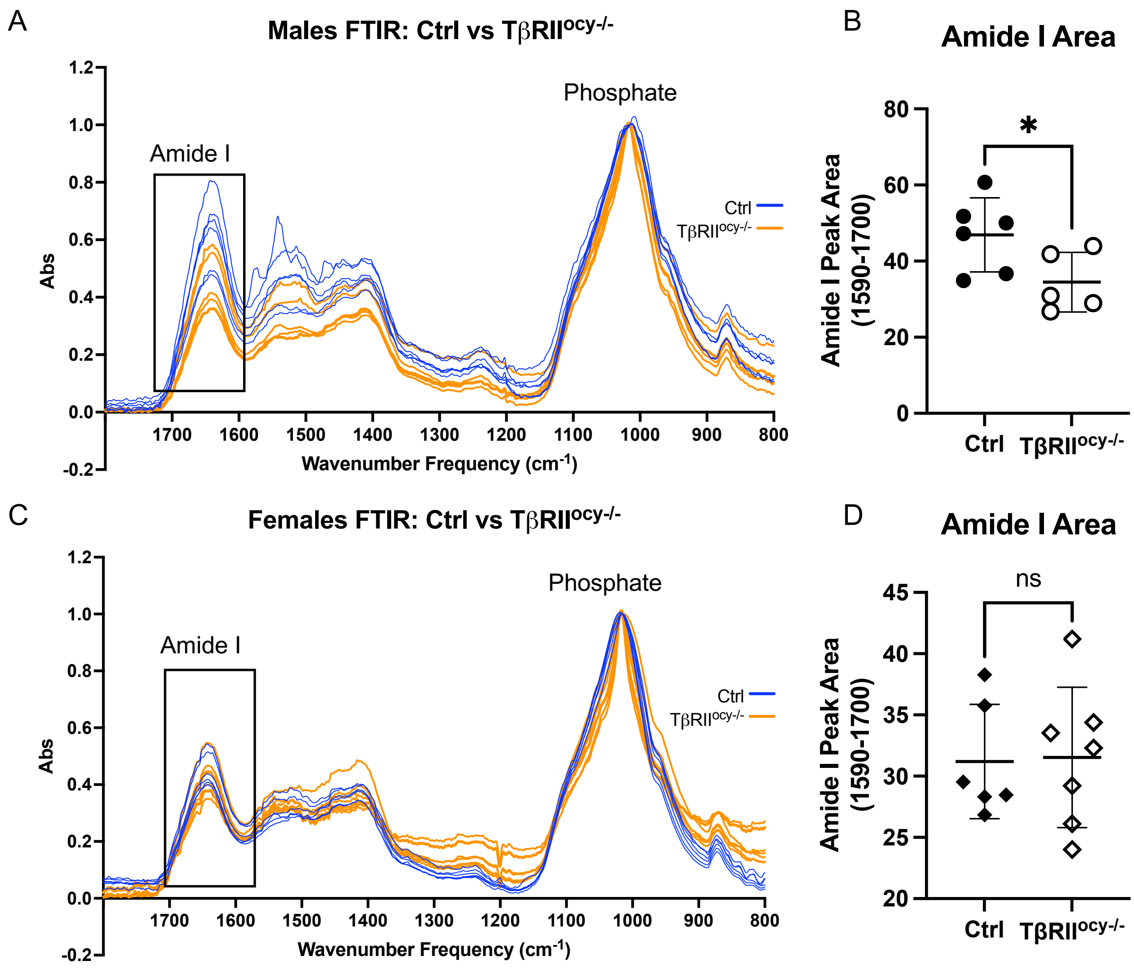

Supplemental Figure 7: Female TβRII$^{ocy-/-}$ proteome regulation. Proteomic analysis of female TβRII$^{ocy-/-}$ femurs (A) via LC-MS identified 1653 proteins each with >2 identified unique peptides. Differential regulation analysis (q-value < 0.05, and absolute Log2(fold-change) > 0.58) identified far fewer proteins than in males, with 11 significantly altered proteins between female TβRII$^{ocy-/-}$ and control bone. Interestingly, female TβRII$^{ocy-/-}$ bone showed significant upregulation (p-value < 0.01 and absolute Log2(normalized fold-change) > 0.58) for PTM sites (B) related to collagen, but these were less numerous than for males.

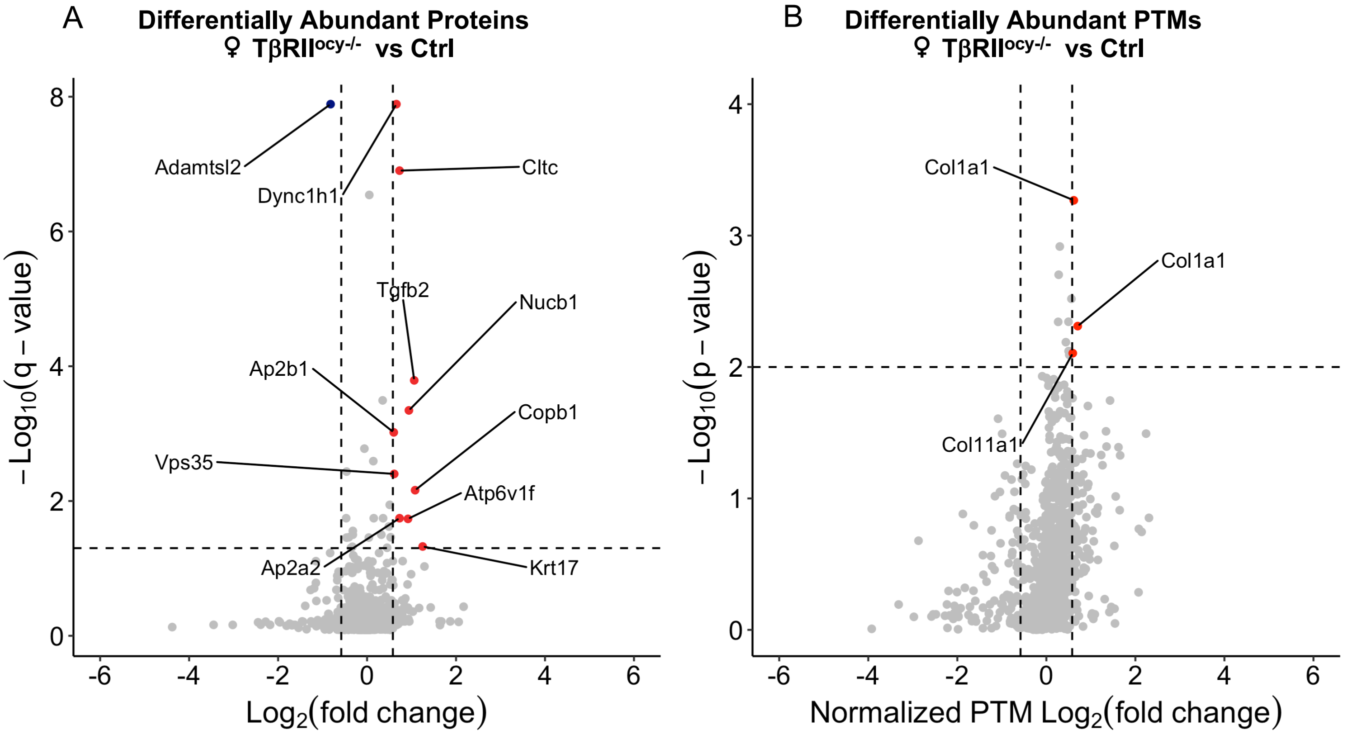

Supplement: Supplementary file 1 — Supplemental Figures [file 41413_2023_303_MOESM1_ESM.pdf]
