# Supplementary material for: Aging impairs the osteocytic regulation of collagen integrity and bone quality
Source: Bone Res. 2024 Feb 26;12:13. doi: 10.1038/s41413-023-00303-7 (PMC10897167; doi:10.1038/s41413-023-00303-7)
Supplement: Supplementary file 2 — Supplemental Extended Methods [file 41413_2023_303_MOESM2_ESM.pdf]

Supplemental Extended Methods

Murine studies

Male C57BL/6 mice (WT, wild type) aged 2 months (N=3), 1 year, 2 years, and 2 ½ years (30 months) (N=4) were received from the Buck Institute for Research on Aging. To interrogate the role of osteocyte-intrinsic TGFβ signaling, mice possessing *loxP* sites flanking exon 4 of the Tgfbr2 gene were crossed with hemizygous −9.6kb-DMP1-Cre$^{+/-}$ mice[1, 2]. In DMP1-Cre$^{+/-}$;TβRII^{flfl} (TβRII$^{ocy-/-}$) mice, the TGFβ receptor II was selectively ablated in osteocytes. After backcrossing this line to a C57BL/6 background, DMP1-Cre$^{-/-}$;TβRII$^{fl/fl}$ (Cre-negative Control) littermates were used as controls. All mice genotypes were confirmed by PCR genotyping. Male and female TβRII$^{ocy-/-}$ and Cre-negative control mice were allowed to age naturally to 4 months (16 weeks), 6 months (24 weeks), 12 months (48 weeks), and 15 months (60 weeks) before euthanasia and sample collection (N=7-9 per group). Over their lifetime, animals were housed in groups no greater than 5 in a specific pathogen-free environment with temperature maintained between 68 °F and 74 °F, humidity between 30% and 70%, with a 12-h light/dark cycle and access ad. libitum to water and rodent chow (LabDiet 5053). All animal procedures were approved by the Institutional Animal Care and Use Committee of the University of California San Francisco and the Buck Institute for Research on Aging.

RNA isolation and analysis of gene expression

Humeri from mice were cleaned of soft tissue with metaphysis and periosteum dissected away, and the marrow removed via centrifugation. The remaining cortical bone of two humeri per mouse were snap-frozen in liquid nitrogen and combined in a single tube with 1.5 mL of QIazol (Invitrogen). While frozen, bone samples in QIazol solution were mechanically homogenized with a rotor-stator homogenizer (GLH, Omni). Lysate was then stored at -80 °C until RNA extraction [3–5].

For RNA extraction, QIAzol lysate was thawed, and 220 µL chloroform per mL of lysate was added and mixed well via pipette. After solution began to separate (2-3 mins on benchtop), samples were then centrifuged for 15 minutes at 12,000 x g at 4°C, and the aqueous supernatant carefully transferred to a clean tube. mRNA was purified using the miRNeasy Mini Kit (Qiagen), following manufacturer's instructions. On-column DNase (Qiagen) was applied for 15 minutes, and the RNA was eluted in 60 µL RNase-free water. RNA concentration was quantified with a NanoDrop spectrophotometer and the product stored at -80 °C.

RNA Sequencing for the aged WT mice (C57BL/6) was completed as described on the Illumina HiSeq 4000 at the UCSF Functional Genomics Core [6]. Single-end 50 bp RNA-seq reads were aligned to the Ensembl mouse GRCm38.87 reference genome using STAR 2.5.2b aligner [7]. Analysis of the aged mice bone resulted in 745 million total reads with an average of 78.7% of these reads aligning uniquely to the mouse genome. The DESeq2 package in R Statistical Computing Environment [8] was used to find differentially expressed genes with a false discovery rate of 0.1. Read count fold change for individual gene comparisons were normalized with reference to young (2 month) WT mice average read count. Statistical comparisons utilized a Bonferroni corrected student's T-test for 6 multiple comparisons among the 4 groups.

For RT-qPCR, RNA was reverse-transcribed using the iScript cDNA synthesis kit (BioRad) for a final concentration of 10 ng cDNA / µl. RT-qPCR of cDNA utilized iQ SYBR Green Supermix (BioRad) with 18s as the housekeeping gene for comparison of target genes Loxl2, loxl3, and Postn **(Supp. Table S1)** on a BioRad Thermocycler (Hercules, CA). In total, 60 ng of cDNA was used per reaction for 18s and ~60ng of cDNA per gene was used for amplification between 20 and 30 cycles. Expression was then quantified by the ΔΔCt method.

TGFβ ELISA

Protein from bone lysates was harvested as previously described[9]. Briefly, for collection of bone specific protein, the distal half of tibia were stripped of periosteum and connective tissue then centrifuged to remove marrow before being snap frozen in liquid nitrogen. Frozen bones were transferred to chilled pre-filled Bead Mill Tubes (ThermoFisher Scientific, Waltham, MA) containing radioimmunoprecipitation buffer [RIPA – 10 mMTris, pH 8, 1 mM EDTA, 1 mM EGTA, 140 mM sodium chloride, 1% sodium pyrophosphate, 100 mM sodium fluoride, 500 µM PMSF, 5 mg/mL eComplete Mini protease inhibitor tablet (Roche, Basil Switzerland)]. Samples were disrupted using a benchtop bead mill homogenizer (Omni International, Kennesaw, GA) at 4°C. Following centrifugation supernatants were sonicated for 8 times (15s/45s rest) in an ice-cold water bath. Serum was collected from blood as described [10]. Protein concentrations were quantified using the Pierce Coomassie (Bradford) Assay Kit (ThermoFisher Scientific, Waltham, MA) before hydrolysis in 6N HCL. Total levels of TGFβ ligand were quantified from hydrolyzed lysates using the Bio-Plex Pro TGF-β Assay Kit (Bio-Rad, Hercules, CA) on a Bio-Plex 200 (Bio-Rad, Hercules, CA). TGFβ ligand levels were normalized to each samples' original total protein concentration.

Histological Analysis

Proximal femurs were stripped of periosteum and connective tissue and fixed for 48 hours in 10% Neutral Buffered Formalin (NBF) and then decalcified in 0.5M disodium and tetrasodium EDTA (pH 7.3-4) for 2 weeks at 4°C and prepared for cryosectioning as described [11–13]. For imaging of large sections of the osteocyte network, femurs stained using the lipophilic dye 1,1'-Dioctadecyl-3,3,3',3'-Tetramethylindocarbocyanine Perchlorate (DiI, ThermoFisher) dissolved in 50% PBS/DMSO and Phalloidin-conjugated AlexaFlor 488 (Invitrogen) for labeling of osteocyte membranes and cytoskeleton, as described [5, 11]. Sections for 3D imaging were taken from the midpoint of the diaphyseal femur halfway between the growth plates. Given the ages and sexes analyzed, the absolute distance changed per animal and so this method was used to obtain consistent regions of interest. Each sample was sectioned at a size of 50 mm, moving from the distal end of the bisected proximal femur midshaft toward the femoral head. Each digitally analyzed region was 35 mm in thickness and excluded ~7.5 mm on either edge of the image stack to avoid imaging artifacts. Histologic TUNEL assays were completed on 8-10 µm sections using the Fluorescein *In Situ* Cell Death Detection Kit from Roche (Basel, Switzerland). Cell death was measured as the percent of identified cell nuclei co-stained with DAPI and Fluorescein against total number of DAPI-positive nuclei. Fluorescent confocal imaging was completed using a Lecia DMi8 (Leica Microsystems, Wetzlar, Germany) inverted confocal microscope running LAS X software, while brightfield imaging was completed with a Nikon Eclipse E800 microscope (Tokyo, Japan). 3D structural

analysis of the osteocyte LCN was completed as described[11]. Briefly, canalicular tortuosity was measured utilizing the Simple Neurite Tracer plug-in for FIJI[14] while Imaris (Oxford Instruments) was used to count individual canaliculi per osteocytes in 3D [13].

μCT

Unfixed femora were scanned using a Scanco μCT50 specimen scanner (Scanco Medical, Wangen-Brüttisellen, Switzerland) with an X-ray potential of 55 kVp, current of 109 μA, and voxel size of 10 μm at room temperature while hydrated in Hanks Balanced Sodium Salts (HBSS, ThermoFisher Scientific, Waltham, MA). Following 3-point bend mechanical testing, distal femur halves containing femoral condyles were scanned again for trabecular bone. Samples displaying damage near the growth plates from the mechanical tests were excluded from trabecular analysis. 3D measurements of cortical and trabecular parameters were obtained using Scanco analysis methods as described [15], while measurements of cortical porosity were collected through manual segmentation and quantification of Scanco generated DICOM files utilizing Dragonfly (Object Research Systems, Montreal, Canada.)

Mechanical Testing

Whole hydrated femurs were loaded to failure in three-point bending in the direction of primary physiological bending (posterior compression) using a Bose Electroforce 3200 test frame with an 8-mm span at a fixed displacement rate of 10 μm/s. Structural parameters were extracted from load-displacement curves using a custom MATLAB script and material properties were acquired by transforming specific structural properties by their individual parameters from μCT as described [16]. Following testing, fracture surface cross-sections were imaged by scanning electron microscopy on a Sigma 500 VP FE-SEM (Zeiss) at an excitation voltage of 15 kV and a partial pressure of 35 Pa. Endosteal and periosteal cross-sectional diameter and thickness were measured with ImageJ and used to calculate moment of inertia assuming an elliptical cross-section for calculation of material properties from structural parameters.

Synchrotron *in situ* Tensile testing

Collagen fibril and hydroxy appetite mineral crystal strains were recorded during uniaxial tension testing of isolated, hydrated ulnae using synchrotron small-angle x-ray scattering (SAXS) and wide-angle x-ray diffraction (WAXD) at beamline 7.3.3 at the Advanced Light Source (LBNL, Berkeley, CA) [17]. This strategy permits simultaneous, real-time measurement of specific strains in the composite materials as compared to bulk tissues as captured by CCD camera and measured with a Digital Image Correlation (DIC) MATLAB package. A two-part dental bonding adhesive (OptiBond, Kerr Corporation, Orange, CA) and resin (Aeliteflo, Bisco Dental, Schaumburg, IL) was used to create a “dumbbell” shape around the epiphyses for clamping without crushing the bone or harming the diaphysis exposed to the X-ray beam. First, the bonding adhesive was applied to the ends of the bone and cured with blue light then the ends were submersed in uncured resin in a 3D printed dumbbell mold and then cured again to solidify the resin around the bone ends. *In situ* tensile tests (N = 7-12 per group) were performed with a TST350 Tensile Testing Stage (Linkam Scientific Inc.) on hydrated samples at RT at a

displacement rate of 2.5 μm/s. The mid-diaphysis of hydrated ulnae were exposed to a perpendicular X-ray beam with spot size 750 μm x 250 μm and an energy of 10 keV for 0.5 s every 5 s until failure or until total radiation dose reached 30 kGy to mitigate effects of radiation exposure on the material performance of the bone [18]. Samples that slipped from clamps, removed from resin prior to fracture, or showed a non-diaphyseal fracture were excluded from final analysis. The changes in collagen *d*-spacing and mineral crystal lattice spacing from the unloaded condition, as seen as a shift in the location of the Bragg scattering peak from SAXS pattern and the Bragg diffraction peak in the WAXD pattern, were used to calculate collagen and mineral associated strains, respectively. Conversion from 2D scattering/diffraction patterns to 1D curves for fitting and extraction of parameters was completed via a custom LabVIEW program [19]. Material specific strains were determined from changes in the d- or lattice spacings over time and were time-matched to their tissue level strain at each point as determined from CCD images of the bone during deformation.

Non-enzymatic Collagen Crosslinking Quantification

Decalcified, mid-diaphyseal femurs were hydrolyzed in HCl (24 hours, 110°C) and fluorescence of neutralized lysates (excitation 370 nm, emission 440 nm) were referenced to a quinine sulfate standard and then normalized to the collagen content calculated from the amount of hydroxyproline, as measured by a colorimetric assay with absorbance at 560nm, for quantification of Advanced Glycation End-products (AGEs) within bone [20].

Fourier-transform Infrared Spectroscopy

Cortical diaphysis from femurs of male and female $T\beta RII^{ocy-/-}$ and littermate controls at 4 months of age (16 weeks) (N=5-7 per group) were dissected and marrow removed via centrifugation. Samples were fixed overnight in 70% ethanol at 4°C prior to an ethanol series for dehydration in 80%/90%/100% ethanol, for 1 hour each at room temperature. Remaining sample moisture was removed via desiccation in a vacuum chamber containing Dri-Rite (Chicago, IL) for 1 hour. Dried cortical bone samples were homogenized by grinding with a polished mortar and pestle to a fine powder. Fourier-transform Infrared (FTIR) spectrums were collected in Attenuated Total Reflection (ATR) mode on a PerkinElmer Spotlight 200i FT-IR Microscopy System (Waltham, MA) made available by the UCSF CCMBM. To account for the possibility of genotype-dependent changes in bone mineral densities, uniform amounts (10 mg) of powdered bone material, enough to cover the ATR crystal, were analyzed per sample. Spectra were background corrected, normalized to mineral content at the peak of phosphate signal (~1010-1030 cm^{-1})[21, 22] and ATR corrected via Spectrum software (PerkinElmer). Prior to normalization, raw intensities of the phosphate peak were compared to ensure that a comparable amount of mineralized bone was analyzed per sample and normalization to this feature would not skew results elsewhere in the collected spectra. Powdered samples were ensured to be in "perfect" contact with the ATR anvil/crystal for collection in ATR mode [23, 24]. Additional grinding and drying was applied if needed. Enzymatic and non-enzymatic collagen quality and crosslinking were quantified by measuring signal intensity at defined locations within the Amide I band [25–27].

Protein Extraction and Mass Spectrometric Quantitative Analysis

Protein Extraction: Femurs had soft tissue and periosteum stripped off, ends dissected at the metaphysis, and the marrow removed via centrifugation. Bones were demineralized by rotating overnight in 1 mL of 1.2 M HCl at 4°C [28]. Bones were subsequently transferred to a new Eppendorf tube and kept on dry ice. All components of the homogenizer including sample tubes and homogenizing plates were cooled using liquid nitrogen. The frozen bones were subsequently pulverized with the SPEX SamplePrep 1600 MiniG tissue homogenizer in polycarbonate tubes with a 9.5 mm steel grinding ball for 2 minutes at 1500 strokes/minute. After the first 2 minutes, the samples were removed from the homogenizer and allowed to cool in liquid nitrogen for 3 minutes and were then homogenized for another 2 minutes at 1500 strokes/minute. Pulverized samples were transferred into a fresh Eppendorf tube using 800 µL of extraction buffer (6 M guanidine hydrochloride GuHCl; 10 mM Tris-HCl; 50 mM EDTA) and incubated by rotating at 4 °C for 72 hrs [29]. Subsequently, the samples were spun for 3 minutes at 15,000 x *g* to pellet bone matrix and the supernatants, containing the proteins, were collected. The supernatant was buffer exchanged to remove GuHCl using Amicon 3 kDa Centrifugal Filters. Samples were washed via spinning through filters at 12,000 x *g* for 20 minutes and resuspended in 500 µL of 10 mM Tris-HCl (pH 7) three times. Final protein aliquots were resuspended in 20 µL 10 mM Tris-HCl. Extracted protein was quantified using a bicinchoninic acid assay (BCA).

Proteolytic Digestion: 50 µg of protein lysate per sample was brought up to 4% SDS using a 10% SDS solution and then reduced using 20 mM dithiothreitol in 50 mM triethylammonium bicarbonate buffer (TEAB, pH 7) at 50 °C for 10 min. Samples were cooled to and held at RT for 10 min, and alkylated using 40 mM iodoacetamide in 50 mM TEAB (pH 7) at RT in the dark for 30 min. Samples were acidified with 12% phosphoric acid to obtain a final concentration of 1.2% phosphoric acid. S-Trap buffer consisting of 90% methanol in 100 mM TEAB at pH ~7.1*,* was added and samples were loaded onto the S-Trap mini spin columns (Protifi, Farmingdale, NY). The entire sample volume was spun through the S-Trap mini spin columns at 4,000 x *g* at RT, binding the proteins to the mini spin columns. Subsequently, S-Trap mini spin columns were washed twice with S-Trap buffer at 4,000 x *g* at RT and placed into clean elution tubes. Samples were incubated for one hour at 47°C with sequencing grade trypsin (Promega, San Luis Obispo, CA) dissolved in 50 mM TEAB (pH 7) at a 1:25 (w/w) enzyme:protein ratio. Finally, additional trypsin solution was added at the same w/w ratio, and proteins were digested overnight at 37°C. Peptides were sequentially eluted from the mini S-Trap spin columns with 50 mM TEAB, 0.5% formic acid (FA) in water, and 50% acetonitrile (ACN) in 0.5% FA. After centrifugal evaporation, samples were resuspended in 0.2% FA in water and desalted with Oasis 10 mg Sorbent Cartridges (Waters, Milford, MA). The desalted elutions were subjected to centrifugal evaporation were re-suspended in 0.2% FA in water at a final concentration of 1 µg/µL. Finally, indexed Retention Time Standards (iRT, Biognosys, Schlieren, Switzerland) were added to each sample according to manufacturer's instructions [30].

Mass Spectrometric Analysis using Data-Independent Acquisitions (DIA): Reverse-phase HPLC-MS/MS analyses were performed in data-independent acquisition (DIA) [31–33] mode on a Dionex UltiMate 3000 system coupled online to an Orbitrap Eclipse Tribrid mass spectrometer (Thermo Fisher Scientific, San Jose, CA). The solvent system consisted of 2% ACN, 0.1% FA in water (solvent A) and 98% ACN, 0.1% FA in water (solvent

B). Digested peptides (200 ng) were loaded onto an Acclaim PepMap 100 C_{18} trap column (0.1 x 20 mm, 5 µm particle size; Thermo Fisher Scientific) over 5 min at 5 µL/min with 100% solvent A. Peptides were eluted on to an Acclaim PepMap 100 C_{18} analytical column (75 µm x 50 cm, 3 µm particle size; Thermo Fisher Scientific) at 300 nL/min using the following gradient (indicated the % of solvent B): 2% B for 5 min, linear from 2% to 20% B in 95 min, linear from 20% to 32% B in 20 min, increase to 80% B in 1 min, hold at 80% B for 9 min, and back to 2% B in 1 min. The column was re-equilibrated for 29 min with 2% of solvent B/98% solvent A, and the total gradient length was 160 min. Each of the biological replicate samples (n=5 for TβRII$^{ocy-/-}$ and 5 for WT) was acquired in data-independent acquisition (DIA) mode [31, 32, 34] in technical duplicates. Survey MS1 spectra were collected at 120,000 resolution (Automatic Gain Control (AGC) target: 3e6 ions, maximum injection time: 60 ms, 350-1,650 *m/z*), and MS2 spectra at 30,000 resolution (AGC target: 3e6 ions, maximum injection time: Auto, Normalized Collision Energy (NCE): 27, fixed first mass 200 *m/z*). The DIA isolation scheme consisted of 26 variable windows covering the 350-1,650 *m/z* range with an overlap of 1 *m/z* per each window **(Supp. Table S2)** [34].

Mass Spectrometric DIA Data Processing: The DIA data files were processed in Spectronaut (version 15.6.211220.50606, Biognosys) using directDIA for both the protein and peptide level. Data was searched against the *Mus musculus* reference proteome with 58,430 entries (UniProtKB-TrEMBL), accessed on 01/31/2018. Data extraction parameters were set as dynamic and non-linear iRT calibration with precision iRT was selected. Trypsin/P was set as the digestion enzyme and two missed cleavages were allowed. Cysteine carbamidomethylation was set as a fixed modification while methionine oxidation and protein N-terminus acetylation were set as dynamic modifications. For the protein level, identification was performed requiring a 1% q-value cutoff on the precursor ion and protein level. Unique protein groups were reported with at least 2 unique peptide identifications. The protein level quantification was based on the peak areas of extracted ion chromatograms (XICs) of 3 – 6 MS2 fragment ions, specifically b- and y-ions, with local normalization and q-value sparse data filtering applied **(Supp. Table S3)**. In addition, iRT profiling was selected. For the post-translational (PTM) hydroxyproline-containing peptide level, proline oxidation was additionally set as variable modification, and identification was performed requiring a 1% q-value cutoff on the precursor ion and protein level. The PTM site localization score was selected with a probability cutoff of 0.75. Quantification was based on XICs of 3 – 6 MS2 fragment ions, specifically b- and y-ions, without normalization as well as data filtering using q-value sparse. Grouping and quantitation of PTM peptides were accomplished using the following criteria: minor grouping by modified sequence and minor group quantity by mean precursor quantity. **(Supp. Table S4)**.

Statistical Analysis and Comparison

Prism v.9 (Graph-pad, San Diego, CA) was used for all statistical comparisons except for ELISA and Proteomic results. Analysis of WT aging male cohorts utilized a one-way ANOVA with an initial alpha of 0.05 with a single factor of age at four different levels (2 mo, 1 yr, 2 yrs, 2 ½ yrs), and post-hoc comparisons used a Bonferroni corrected alpha of 0.008 (0.05 / 6) for differences in transcriptional activity across the 4 age groups compared.

Two-Way ANOVA comparisons for TβRII$^{ocy-/-}$ and Cre-negative controls used factors of age and genotype. Significance (p) values for all interaction terms and main effects of age and genotype can be found in **Supplemental Table S5**. For Two-Way ANOVA analyses, factors of age and genotype were considered at an alpha level of 0.05. While sex differences are noted throughout, they were not statistically compared to each other due to their difference in biologic mechanism and each sex was run in independent Two-Way ANOVA comparisons. Within each Two-Way ANOVA, age had up to three independent levels (4, 12 and 15 months depending on ages included) and genotype had 2 (Cre-negative control, TβRII$^{ocy-/-}$). *A priori* planned pairwise post hoc comparisons were completed with the Fisher's Least Significance Difference Test that does not correct p-value for multiple comparisons, and instead the significant level (alpha = 0.05) was Bonferroni corrected for the number of comparisons being made. In a planned manner, not all possible pairwise comparisons are scientifically meaningful and power was not sacrificed for excessive comparisons. In post hoc analysis for factors that violated the Fisher's LSD assumption that all groups are sampled from populations with the same standard deviation, standard Student's T-tests were completed for post hoc assessment of significance. Because ELISA analysis had additional group complexity (age, sex, genotype, tissue source, and ligand sub-type), full ensemble results were first analyzed with univariable and multilevel Linear Mixed Models of the log of concentration to determine what factors most strongly predicted ligand concentration **(Supplemental Table S6)**. Afterward, pairwise comparisons were completed with the non-parametric Wilcoxon signed-rank test with significant alpha values Bonferroni corrected for the specific number of comparisons being completed in each pairwise comparison. Synchrotron tissue vs material strain regressions were completed with a linear least squares method to generate linear slopes for statistical comparisons via an extra sum-of-squares F test that compares independent fits of a pair of regressions with a global fit. Regressions were considered statistically different if the p-value for the comparison of slopes of independent regressions to the global fit was below alpha = 0.05/4 = 0.0125 for a 4-way Bonferroni correction for multi-comparisons. P-values considered 'trending' between $p<0.1$ and Bonferroni corrected alphas are noted on figures.

Differential expression analyses of mouse femur proteomes were performed using a paired t-test, and p-values were corrected for multiple testing using the Storey method [35]. Specifically, group wise testing corrections were applied to obtain q-values. For the protein level, protein groups with at least two unique peptides, q-value < 0.05, and absolute Log2(fold-change) > 0.58 were considered significantly-altered **(Supp. Table S3)**. For the quantification of the hydroxyproline-containing peptides, post translational modification (PTM) fold-change values were normalized by the corresponding protein fold-change values, and hydroxyproline-containing peptides with p-value < 0.01 and absolute Log2(normalized fold-change) > 0.58 were considered significantly-altered **(Supp. Table S4)**. Volcano plots were generated using the ggplot2 package [36] in R (version 4.0.5; RStudio, version 1.4.1106).

References

1. Lu Y, Xie Y, Zhang S, et al (2007) DMP1-targeted Cre expression in odontoblasts and osteocytes. J Dent Res 86:320–325. https://doi.org/10.1177/154405910708600404

2. P. L, J. L, M. E, et al (2002) Induced disruption of the transforming growth factor beta type II receptor gene in mice causes a lethal inflammatory disorder that is transplantable. Blood 100:560–8. https://doi.org/10.1182/blood.v100.2.560

3. Dole NS, Mazur CM, Acevedo C, et al (2017) Osteocyte-Intrinsic TGF-β Signaling Regulates Bone Quality through Perilacunar/Canalicular Remodeling. Cell Rep 21:2585–2596. https://doi.org/10.1016/j.celrep.2017.10.115

4. Fowler TW, Acevedo C, Mazur CM, et al (2017) Glucocorticoid suppression of osteocyte perilacunar remodeling is associated with subchondral bone degeneration in osteonecrosis. Sci Rep 7:44618. https://doi.org/10.1038/srep44618

5. Yee CS, Schurman CA, White CR, Alliston T (2019) Investigating Osteocytic Perilacunar/Canalicular Remodeling. Curr Osteoporos Rep 17:157–168. https://doi.org/10.1007/s11914-019-00514-0

6. Kaya S, Schurman CA, Dole NS, et al (2022) Prioritization of Genes Relevant to Bone Fragility Through the Unbiased Integration of Aging Mouse Bone Transcriptomics and Human GWAS Analyses. J Bone Miner Res 37:804–817. https://doi.org/10.1002/jbmr.4516

7. Dobin A, Davis CA, Schlesinger F, et al (2012) STAR: ultrafast universal RNA-seq aligner. Bioinformatics 29:15–21. https://doi.org/10.1093/bioinformatics/bts635

8. Love MI, Huber W, Anders S (2014) Moderated estimation of fold change and dispersion for RNA-seq data with DESeq2. Genome Biol 15:31. https://doi.org/10.1186/s13059-014-0550-8

9. Nguyen J, Massoumi R, Alliston T (2019) CYLD, a mechanosensitive deubiquitinase, regulates TGFβ signaling in load-induced bone formation. Bone 131:115148. https://doi.org/10.1016/j.bone.2019.115148

10. Dole NS, Yee CS, Mazur CM, et al (2020) TGFβ regulation of perilacunar/canalicular remodeling is sexually dimorphic. J Bone Mineral Res Official J Am Soc Bone Mineral Res. https://doi.org/10.1002/jbmr.4023

11. Dole NS, Yee CS, Schurman CA, et al (2021) Assessment of Osteocytes: Techniques for Studying Morphological and Molecular Changes Associated with Perilacunar/Canalicular Remodeling of the Bone Matrix. Methods Mol Biol 2230:303–323. https://doi.org/10.1007/978-1-0716-1028-2_17

12. Tiede-Lewis LM, Xie Y, Hulbert MA, et al (2017) Degeneration of the osteocyte network in the C57BL/6 mouse model of aging. Aging 9:2190–2208. https://doi.org/10.18632/aging.101308

13. Schurman CA, Verbruggen SW, Alliston T (2021) Disrupted Osteocyte Connectivity and Pericellular Fluid Flow in Bone with Aging and Defective TGF-β Signaling. Proc Natl Acad Sci USA 118:e2023999118. https://doi.org/10.1073/pnas.2023999118

14. Schindelin J, Arganda-Carreras I, Frise E, et al (2012) Fiji: an open-source platform for biological-image analysis. Nat Methods 9:676–682. https://doi.org/10.1038/nmeth.2019

15. Bouxsein ML, Boyd SK, Christiansen BA, et al (2010) Guidelines for assessment of bone microstructure in rodents using micro–computed tomography. J Bone Miner Res 25:1468–1486. https://doi.org/10.1002/jbmr.141

16. Jepsen KJ, Silva MJ, Vashishth D, et al (2015) Establishing Biomechanical Mechanisms in Mouse Models: Practical Guidelines for Systematically Evaluating Phenotypic Changes in the Diaphyses of Long Bones. J Bone Miner Res 30:951–966. https://doi.org/10.1002/jbmr.2539

17. Hexemer1 A, Bras2 W, Glossinger1 J, et al (2010) A SAXS/WAXS/GISAXS Beamline with Multilayer Monochromator. J Phys Conf Ser 247:012007. https://doi.org/10.1088/1742-6596/247/1/012007

18. Barth HD, Zimmermann EA, Schaible E, et al (2011) Characterization of the effects of x-ray irradiation on the hierarchical structure and mechanical properties of human cortical bone. Biomaterials 32:8892–8904. https://doi.org/10.1016/j.biomaterials.2011.08.013

19. Zimmermann EA, Schaible E, Bale H, et al (2011) Age-related changes in the plasticity and toughness of human cortical bone at multiple length scales. Proceedings of the National Academy of Sciences 108:14416–14421. https://doi.org/10.1073/pnas.1107966108

20. Acevedo C, Sylvia M, Schaible E, et al (2018) Contributions of Material Properties and Structure to Increased Bone Fragility for a Given Bone Mass in the UCD-T2DM Rat Model of Type 2 Diabetes. J Bone Miner Res 27:219–1075. https://doi.org/10.1002/jbmr.3393

21. Imbert L, Gourion-Arsiquaud S, Villarreal-Ramirez E, et al (2018) Dynamic structure and composition of bone investigated by nanoscale infrared spectroscopy. Plos One 13:e0202833. https://doi.org/10.1371/journal.pone.0202833

22. Paschalis EP, Mendelsohn R, Boskey AL (2011) Infrared Assessment of Bone Quality: A Review. Clin Orthop Relat Res 469:2170–2178. https://doi.org/10.1007/s11999-010-1751-4

23. Diem M (2018) Modern Vibrational Spectroscopy and Micro-Spectroscopy. https://doi.org/10.1002/9781118824924

24. Ruysschaert J-M, Raussens V (2018) Peptide Self-Assembly, Methods and Protocols: ATR-FTIR Analysis of Amyloid Proteins. Methods Mol Biology 1777:69–81. https://doi.org/10.1007/978-1-4939-7811-3_3

25. Paschalis EP, Gamsjaeger S, Tatakis DN, et al (2015) Fourier Transform Infrared Spectroscopic Characterization of Mineralizing Type I Collagen Enzymatic Trivalent Cross-Links. Calcified Tissue Int 96:18–29. https://doi.org/10.1007/s00223-014-9933-9

26. Paschalis EP, Verdelis K, Doty SB, et al (2001) Spectroscopic Characterization of Collagen Cross-Links in Bone. J Bone Miner Res 16:1821–1828. https://doi.org/10.1359/jbmr.2001.16.10.1821

27. Schmidt FN, Zimmermann EA, Campbell GM, et al (2017) Assessment of collagen quality associated with non-enzymatic cross-links in human bone using Fourier-transform infrared imaging. Bone 97:243–251. https://doi.org/10.1016/j.bone.2017.01.015

28. Jiang X, Ye M, Jiang X, et al (2007) Method Development of Efficient Protein Extraction in Bone Tissue for Proteome Analysis. J Proteome Res 6:2287–2294. https://doi.org/10.1021/pr070056t

29. Creecy A, Brown KL, Rose KL, et al (2021) Post-Translational Modifications in Collagen Type I of Bone in a Mouse Model of Aging. Bone 143:115763. https://doi.org/10.1016/j.bone.2020.115763

30. Escher C, Reiter L, MacLean B, et al (2012) Using iRT, a normalized retention time for more targeted measurement of peptides. Proteomics 12:1111–1121. https://doi.org/10.1002/pmic.201100463

31. Gillet LC, Navarro P, Tate S, et al (2012) Targeted Data Extraction of the MS/MS Spectra Generated by Data-independent Acquisition: A New Concept for Consistent and Accurate Proteome Analysis*. Mol Cell Proteomics 11:O111.016717. https://doi.org/10.1074/mcp.o111.016717

32. Collins BC, Hunter CL, Liu Y, et al (2017) Multi-laboratory assessment of reproducibility, qualitative and quantitative performance of SWATH-mass spectrometry. Nat Commun 8:291. https://doi.org/10.1038/s41467-017-00249-5

33. Schilling B, Gibson BW, Hunter CL (2017) Proteomics, Methods and Protocols. Methods Mol Biology 1550:223–233. https://doi.org/10.1007/978-1-4939-6747-6_16

34. Bruderer R, Bernhardt OM, Gandhi T, et al (2017) Optimization of Experimental Parameters in Data-Independent Mass Spectrometry Significantly Increases Depth and Reproducibility of Results*. Mol Cell Proteomics 16:2296–2309. https://doi.org/10.1074/mcp.ra117.000314

35. Burger T (2018) Gentle Introduction to the Statistical Foundations of False Discovery Rate in Quantitative Proteomics. J Proteome Res 17:12–22. https://doi.org/10.1021/acs.jproteome.7b00170

36. Wickham H (2016) ggplot2: Elegant Graphics for Data Analysis, 1st ed. Springer-Verlag, New York, NY
